# Circular RNA MKLN1 promotes epithelial-mesenchymal transition in pulmonary fibrosis by regulating the miR-26a/b-5p/CDK8 axis in human alveolar epithelial cells and mice models

**DOI:** 10.1007/s00204-024-03700-x

**Published:** 2024-03-09

**Authors:** Yong Zhu, Xiaoxiao Meng, Xian Zhu, Jiaxiang Zhang, Hui lv, Feiyao Wang, Jinfeng Wang, Cheng Chen, Mengting Chen, Dapeng Wang, Wei Jin, Rui Tian, Ruilan Wang

**Affiliations:** 1 Department of Critical Care Medicine, School of Medicine, Shanghai General Hospital, Shanghai Jiaotong University, 650 Xinsongjiang Road, Shanghai, 201620 China; 2 Department of Intensive Care Medicine, Wuxi People’s Hospital, Nanjing Medical University, Wuxi, 214021 Jiangsu China

**Keywords:** Pulmonary fibrosis, Circular RNA MKLN1, Epithelial-mesenchymal transition, miR-26a/b, CDK8

## Abstract

Pulmonary fibrosis involves destruction of the lung parenchyma and extracellular matrix deposition. Effective treatments for pulmonary fibrosis are lacking and its pathogenesis is still unclear. Studies have found that epithelial-mesenchymal transition (EMT) of alveolar epithelial cells (AECs) plays an important role in progression of pulmonary fibrosis. Thus, an in-depth exploration of its mechanism might identify new therapeutic targets. In this study, we revealed that a novel circular RNA, MKLN1 (circMKLN1), was significantly elevated in two pulmonary fibrosis models (intraperitoneally with PQ, 50 mg/kg for 7 days, and intratracheally with BLM, 5 mg/kg for 28 days). Additionally, circMKLN1 was positively correlated with the severity of pulmonary fibrosis. Inhibition of circMKLN1 expression significantly reduced collagen deposition and inhibited EMT in AECs. EMT was aggravated after circMKLN1 overexpression in AECs. MiR-26a-5p/miR-26b-5p (miR-26a/b), the targets of circMKLN1, were confirmed by luciferase reporter assays. CircMKLN1 inhibition elevated miR-26a/b expression. Significantly decreased expression of CDK8 (one of the miR-26a/b targets) was observed after inhibition of circMKLN1. EMT was exacerbated again, and CDK8 expression was significantly increased after circMKLN1 inhibition and cotransfection of miR-26a/b inhibitors in AECs. Our research indicated that circMKLN1 promoted CDK8 expression through sponge adsorption of miR-26a/b, which regulates EMT and pulmonary fibrosis. This study provides a theoretical basis for finding new targets or biomarkers in pulmonary fibrosis.

**Supplementary Information:**

The online version contains supplementary material available at 10.1007/s00204-024-03700-x.

## Introduction

Pulmonary fibrosis is the end stage of various interstitial lung diseases characterized by destruction of the lung parenchyma and deposition of extracellular matrix. In recent years, the incidence of pulmonary fibrosis has been increasing yearly, with a poor prognosis and a median survival period of 3–5 years after diagnosis. Pulmonary fibrosis has seriously impaired the quality of life of patients and has become a major public health problem (Chanda et al. 2019). This condition has a complex etiology and is usually caused by persistent exposure of the respiratory tract to microorganisms, respirable atmospheric particles, irritants, drugs, poisons, pollutants, allergens, and pathogens that cause inflammation in the lungs, destroying alveolar epithelial cells (AECs) and resulting in secretion of high levels of inflammatory factors (Raghu et al. 2022; Thannickal et al. 2004). Although some antifibrotic drugs (nintedanib and pirfenidone) can slow the progression of the disease, they still do not significantly reduce patient mortality. The pathogenesis of pulmonary fibrosis is still unclear (Canestaro et al. 2016). AECs have been confirmed as a central factor in the progression of pulmonary fibrosis due to a decrease in their own repair function in a sustained microinjury environment, leading to myofibroblast accumulation (Parimon et al. 2020). Under specific physiological or pathological conditions, epithelial cells can develop mesenchymal cell morphology, resulting in loss of the original cell polarity, reduced adhesion and cytoskeletal changes, thus enhancing cell migration and motility; this process is called epithelial-mesenchymal transition (EMT). EMT plays an important role in pathological states, especially in inflammation, organ fibrosis and highly metastatic cancers (Chen et al. 2017; Nieto et al. 2016). Therefore, in-depth research on the mechanism of EMT occurrence might be important for finding new targets for the treatment of pulmonary fibrosis.

Circular RNA (circRNA) is a new type of endogenous noncoding RNA molecule with a closed loop structure, consisting mainly of exons and/or introns, that is commonly found in eukaryotic cells. CircRNAs are highly stable, species-conserved, and cell- and tissue-specific molecules (Qu et al. 2015). Increasing studies have examined the role of circRNAs in lung diseases such as lung cancer, acute respiratory distress syndrome and pulmonary hypertension (Wang et al. 2019). Tan et al*.* recently found that circRNA F-circEA could promote the proliferation and migration of tumor cells in non-small cell lung cancer (Tan et al. 2018). Li et al*.* used gene chips to detect circRNA expression in the plasma of idiopathic pulmonary fibrosis (IPF) patients, which showed elevated expression of three circRNAs (hsa_circRNA_100906, hsa_circRNA_102100, hsa_circRNA_102348) and decreased expression of three circRNAs (hsa_circRNA_101225, hsa_circRNA_104780, hsa_circRNA_101242). Bioinformatics analysis revealed that these circRNAs may be involved in regulating the expression of key factors in fibrosis formation (R. Li et al. 2018a, b). The above results suggest that circRNAs may play an important role in the development of pulmonary fibrosis. However, the explicit functions and mechanisms of circRNAs in pulmonary fibrosis are still unclear.

CircRNAs function mainly by competitively binding (adsorbing) microRNAs (miRNAs) and blocking the repressive effects of miRNAs on their downstream target genes, also known as miRNA sponges (Kristensen et al. 2018; Li et al. 2018a, b). The circRNA CDR1as (CIRS-7) is one of the most studied circRNAs. CIRS-7 was shown to act as a sponge for miR-7 in the SiO_2_-induced lung fibrosis model. CIRS-7 is an important fibrosis inhibitor that targets the transforming growth factor-β (TGF-β)/Smad signaling pathway and inhibits endothelial transformation (Yao et al. 2018). In a bleomycin (BLM)-induced mouse lung fibrosis model in our previous study, circHIPK3 expression was upregulated in fibroblast-to-myofibroblast transition (FMT)-derived myofibroblasts. CircHIPK3 regulates FMT by acting as an endogenous miR-338-3p sponge and increasing Sox4 and COL1A1 expression (Zhang et al. 2019). The expression of hsa_circ_0044226 was significantly higher in the lung tissues of IPF patients than in those of healthy controls. Targeted knockdown of hsa_circ_0044226 inhibited pulmonary fibrosis, possibly by inhibiting the expression of CDC27, which in turn prevented EMT progression (Qi et al. 2020). However, the expression profile of circRNAs in AECs and key regulatory mechanisms in EMT have not been reported.

In this study, we analyzed the expression profile of circRNAs in two models of AECs [TGF-β1 and paraquat (PQ)]. We identified a novel circRNA, MKLN1 (circMKLN1), that was significantly elevated during EMT. CircMKLN1 expression was also significantly elevated in lung fibrosis tissues, and knockdown of circMKLN1 expression significantly inhibited BLM- and PQ-induced lung fibrosis progression. Further exploration of the mechanism showed that circMKLN1 promoted CDK8 expression through competitive binding of miR-26a-5p/miR-26b-5p (miR-26a/b), thereby promoting EMT. Therefore, this study expands and deepens the understanding of the role and mechanism of circMKLN1 in pulmonary fibrosis, and circMKLN1 may become a promising target for the treatment of pulmonary fibrosis.

## Methods and materials

### Cell culture and transfection

Human type II AECs from normal lung tissues were difficult to culture in vitro and were quickly transformed into type I AECs. A human lung cancer cell line (A549) has the physiological activity and biological functions of type II AECs with stable and reproducible performance and has been widely used in the study of lung diseases (Santos et al. 2004; Foster et al. 1998). A549 cells were cultured with F-12 K medium (containing 10% fetal bovine serum [Gibco, Cat.No.: 2553997CP)] and 5% CO_2_ at 37 °C. The cells were changed every 2 d until they reached 70–80% confluency and were then ready for experiments. For transfection, A549 cells in the logarithmic growth phase were selected and inoculated in 6-well plates. CircMKLN1 overexpression plasmids (Genomeditech, 2 μg of each well), circMKLN1 siRNA, miR-26a/b mimics or inhibitors (GenePharma, 100 pmol of each well) were transfected into cells with Lipofectamine 2000 (Thermo Fisher Scientific, Inc., Cat.No.: 11668019) according to the manufacturer’s instructions. Cells were given subsequent stimulation and other treatments 48 h after transfection. Cells stimulated with a final concentration of 800 μM PQ (Sigma‒Aldrich, Cat.No.: 36541) and a final concentration of 5 ng/ml TGF-β1 (MedChemExpress, Cat.No.: HY-P7118) were used. Sequences of siRNA, mimics and inhibitors are shown in Table S1.

### Analysis of the circRNA expression profile

The A549 cells were cultured and substratified by applying PQ stimulation for 24 h and TGF-β1 stimulation for 48 h. The cells were collected for high-throughput sequencing and divided into the control group, PQ group, and TGF-β1 group. The basic procedure of high-throughput sequencing was as follows: extract total RNA from each group, remove ribosomal RNA from the total RNA, remove linear RNA from the samples using RNase R, synthesize cDNA by reverse transcription after fragmentation of RNA, amplify with PCR after end repair and addition of splice primers, screen the library of a suitable size by quality control, and finally sequence on the machine. After the raw sequencing data were obtained, the data were initially filtered, and the circRNAs were identified according to the principles reported in the literature (Zhang et al. 2014). The detection and preliminary data analysis of circRNA high-throughput sequencing was performed by Guangzhou RiboBio Co., Ltd.

### Animal experiments

C57BL/6 mice (6–8 weeks old) of SPF grade were randomly divided into the control, PQ and BLM (Selleckchem, Cat.No.: S1214) groups. After the mice were anesthetized with sodium pentobarbital, the PQ group was immediately injected intraperitoneally with PQ (50 mg/kg), and the BLM group was injected intratracheally with BLM (5 mg/kg) to establish a fibrosis model. In the PQ group, the mice were sacrificed on days 1, 3, and 7, and lung tissues were retained. In the BLM group, the mice were sacrificed on day 28, and lung tissues were retained. CircMKLN1 AAV-shRNA was constructed (Genomeditech), and the control reagent suspension (30 μl per nostril) was slowly introduced dropwise through the inner wall of the nostrils of the mice. Then, the mice were kept for 2 weeks. The treated mice were randomly divided into the control group, PQ group, PQ + circMKLN1 AAV-shRNA group, BLM group, and BLM + circMKLN1 AAV-shRNA group. PQ and BLM mouse models were constructed according to the above method. Mice in the PQ group were killed on day 7 after modeling, and mice in the BLM group were sacrificed on day 28 after modeling (5 mice in each group). After removing the complete lung organs of mice, saline was injected from the trachea to rinse the lung tissues. Then the half of left lung lobe was fixed in 4% formaldehyde solution, dehydrated, made transparent, wax-impregnated and embedded to make paraffin sections; the remaining lung tissues were stored at −80 ℃. All animal feeding and experimental operations were approved by the ethics committee of Shanghai General Hospital.

### Histopathological examination of lung tissues

The mouse and human lung tissues were fixed in 4% paraformaldehyde, embedded in paraffin wax and cut into 3 µm thick sections. These sections were stained for HE, Masson and Sirius scarlet according to the instructions. Then, these sections were observed by microscopy, and images were collected.

### RNA extraction and qRT‒PCR

Treated A549 cells and mouse lung tissues were collected. Total RNA was extracted according to the instructions of the TRIzol kit (Thermo Fisher Scientific, Inc., Cat.No.: 15596026). One microgram of RNA samples was reverse transcribed using the reverse transcription kit (PrimeScript^™^ RT Master Mix, TaKaRa, Cat.No.: RR036A), and the cDNA products were amplified by PCR in an Applied Biosystems QuantStudio 6 Flex system (Life Technologies) using the SYBR Green^™^ Premix Ex Taq^™^ II kit (TaKaRa, Cat.No.: RR420A) with the following reagents: 6 μL of RNase-free water, 10 μL of SYBR Green Premix Ex Taq II, 0.5 μL of ROX Reference Dye II, and 0.5 μL of RNA from the control group. The PCR conditions were as follows: 95 ℃ (30 s) predenaturation; 95 ℃ (5 s) denaturation; 95 ℃ (10 s) → 60 ℃ (30 s) annealing and extension, 40 cycles; 95 ℃ (15 s) → 60 ℃ (1 min) → 95 ℃ (15 s). The reference gene β-actin was used as the internal control. RNA expression was obtained by the 2^−ΔΔCt^ algorithm based on the Ct values of the samples. The primers of miR-26a (Cat.No.: MQPS0000887-1-100), miR-26b (Cat.No.: MQPS0000889-1-100), miR-22 (Cat.No.: MQPS0000861-1-100), miR-432 (Cat.No.: MQPS0001331-1-100), miR-579 (Cat.No.: MQPS0001912-1-100), and miR-6807 (Cat.No.: MQPSCM001-1) were obtain from Guangzhou RiboBio Co., Ltd. The sequences of other primers [Sangon Biotech (Shanghai) Co., Ltd.] for each target gene are shown in Table S2.

### Western blotting

The treated cells and lung tissues of each group were lysed with protein lysis solution for 30 min in an ice bath, and the total protein was extracted. Proteins were taken for quantitative analysis by a BCA protein quantification kit (Beyotime Biotechnology, Cat.No.: P0010), and the separated protein samples was transferred to a PVDF membrane by SDS⁃PAGE (Beyotime Biotechnology, Cat.No.: P0012A and P0562) gel electrophoresis at 100 V for 1.5 h. The membrane was transferred at 300 mA for 1.5 h. After the membrane was blocked with 5% skim milk for 2 h, primary antibodies targeting E-cadherin (1:1000, Abcam, Cat.No.: ab184633), α-SMA (1:1000, Abcam, Cat.No.: ab7817), vimentin (1:1000, ﻿Cell Signaling Technology, Cat.No.: 5741), CDK8 (1:500, Abcam, Cat.No.: ab224828) or β-actin (1:1000, Beyotime Biotechnology, Cat.No.: AF0003) were added and incubated overnight at 4 ℃, followed by 2 ~ 3 washes with TBST. A secondary antibody labeled with HRP (1:2000, Beyotime Biotechnology, Cat.No.: A0208 and A0216) was added and incubated at room temperature for 2 h. The luminescence reaction was performed by adding ECL (Millipore, Cat.No.: WBULP) reaction solution and observed and photographed under a chemiluminescence instrument.

### Fluorescence in situ hybridization (FISH)

The FISH probes of circMKLN1 and miR-26a/b were gained from GenePharma. A549 cell crawls, mouse lung tissues and lung tissues from patients with pulmonary fibrosis were collected. The lung tissue samples were routinely washed, dehydrated, fixed in paraffin, and cut into 5-μm-thick sections. The sections were routinely dehydrated in gradients of 70%, 85% and 100% ethanol, digested with 20 μg/mL proteinase K for 5 min, and washed three times with PBS (3 min/wash); the prehybridization solution was incubated for 1 h at 37 ℃. The next day, the hybridization solution was discarded, and the samples were washed with 2 × sodium citrate buffer (SSC) at 37 ℃ for 10 min, 1 × SSC at 37 ℃ for 5 min and 0. 5 × SSC at room temperature for 10 min. The A549 cell crawls were fixed with 4% paraformaldehyde for 20 min and washed three times with PBS. And then the A549 cell crawls were treated from the digested step as above. These slices were then sealed with antifluorescence quenching sealer, placed under a fluorescence microscope and photographed. The probe sequences which used in FISH was shown in Table S3.

### Immunofluorescence

A549 cells were inoculated in a 6-well plate covered with coverslips, and the cells were treated accordingly according to the study purpose. The cells were washed, fixed in 4% paraformaldehyde for 30 min, perforated with 0.1% Triton X-100 (Sigma-Aldrich, Cat.No.: 93443) for 10 min at room temperature, and blocked with 5% BSA (Beyotime Biotechnology, Cat.No.: ST023) for 1 h. E-cadherin (1:100) and α-SMA (1:100) primary antibodies were incubated overnight at 4 ℃, and the corresponding fluorescent secondary antibodies (1:200, Beyotime Biotechnology, Cat.No.: A0428 and A0473) were used the next day for 1.5 h at room temperature and protected from light. The nuclei were then restained with DAPI (Beyotime Biotechnology, Cat.No.: C1005) for 5 min, and finally, the slides were sealed with antifluorescent bursting agent on microscope slides. The images were collected by laser scanning confocal microscopy (Leica TCS SP8, Leica).

### Luciferase reporter assay

Based on the predicted potential miR-26a/b binding site on circMKLN1, a dual-luciferase reporter plasmid, PGL3-circMKLN1 WT, containing this potential binding sequence was constructed, and the recombinant plasmid PGL3-circMKLN1 MT with mutation of this potential binding sequence was prepared (Genomeditech). One microgram of recombinant luciferase reporter plasmid, 1.5 ng of pRLTK [which can express sea kidney luciferase (RL) and serve as an internal reference plasmid], 100 nmol/L miR-26a/b mimic, etc., were transfected into HEK293 cells at 70% confluence, and the intensity of firefly luciferase (FL) and RL was measured after 24 h. The intensity of FL and RL was measured, and the binding between circMKLN1 and miR-26a/b was determined based on the FL/RL ratio. In addition, the dual luciferase reporter plasmid PGL3-CDK8 WT and mutant recombinant plasmid PGL3-CDK8 MT were constructed, and the binding between miR-26a/b and CDK8 was verified by applying the above method.

### RNase R and actinomycin D treatment

The RNase R reaction solution was prepared according to the instructions of RNase R reagent (Epicentre Technologies, Cat.No.: RNR07250), RNA was digested at 37 ℃ for 15 min, and RNase R was inactivated by incubation at 70 ℃ for 10 min. qRT⁃PCR assays were performed to detect the expression levels of circMKLN1 and MKLN1. After treatment of the cells with 2 μg/mL actinomycin D (Sigma-Aldrich, Cat.No.: SBR00013) for 0, 12 and 24 h, total cellular RNA was extracted according to the above method, and the expression levels of circMKLN1 and MKLN1 were measured by qRT⁃PCR.

### Cytoplasmic and nuclear fractionation assay

The nucleus and cytoplasm of A549 cells were isolated and purified using the Paris Kit (Thermo Fisher Scientific, Cat.No.: AM1921) according to the instructions. Total RNA was isolated from the nucleus and cytoplasm using TRIzol reagent. qRT-PCR was then performed to detect the distribution of circMKLN1 in A549 cells. β-actin and U6 were used as positive controls for the cytoplasmic and nuclear fractions, respectively.

### Clinical samples

The clinical samples collected were approved by the ethics committees of Shanghai General Hospital and Wuxi People’s Hospital. In this research, 5 normal lung tissues were obtained from the noncancerous lung areas of patients with lung cancer. Five fibrotic lung tissues were obtained from patients with IPF at lung transplantation. All clinical samples were fixed in formalin solution for subsequent experiments.

### Statistical analysis

GraphPad Prism 9 was used for graphing, and SPSS 21.0 was used for statistical analysis. Experimental data are expressed as the mean ± standard deviation. A t test was used for comparison of two independent samples, and one-way ANOVA was used for comparisons among multiple groups. p < 0.05 was considered a statistically significant difference. The results were reproduced at least in three independent experiments.

## Results

### CircRNA expression profile in AECs by high-throughput sequencing

**Fig. 1:**
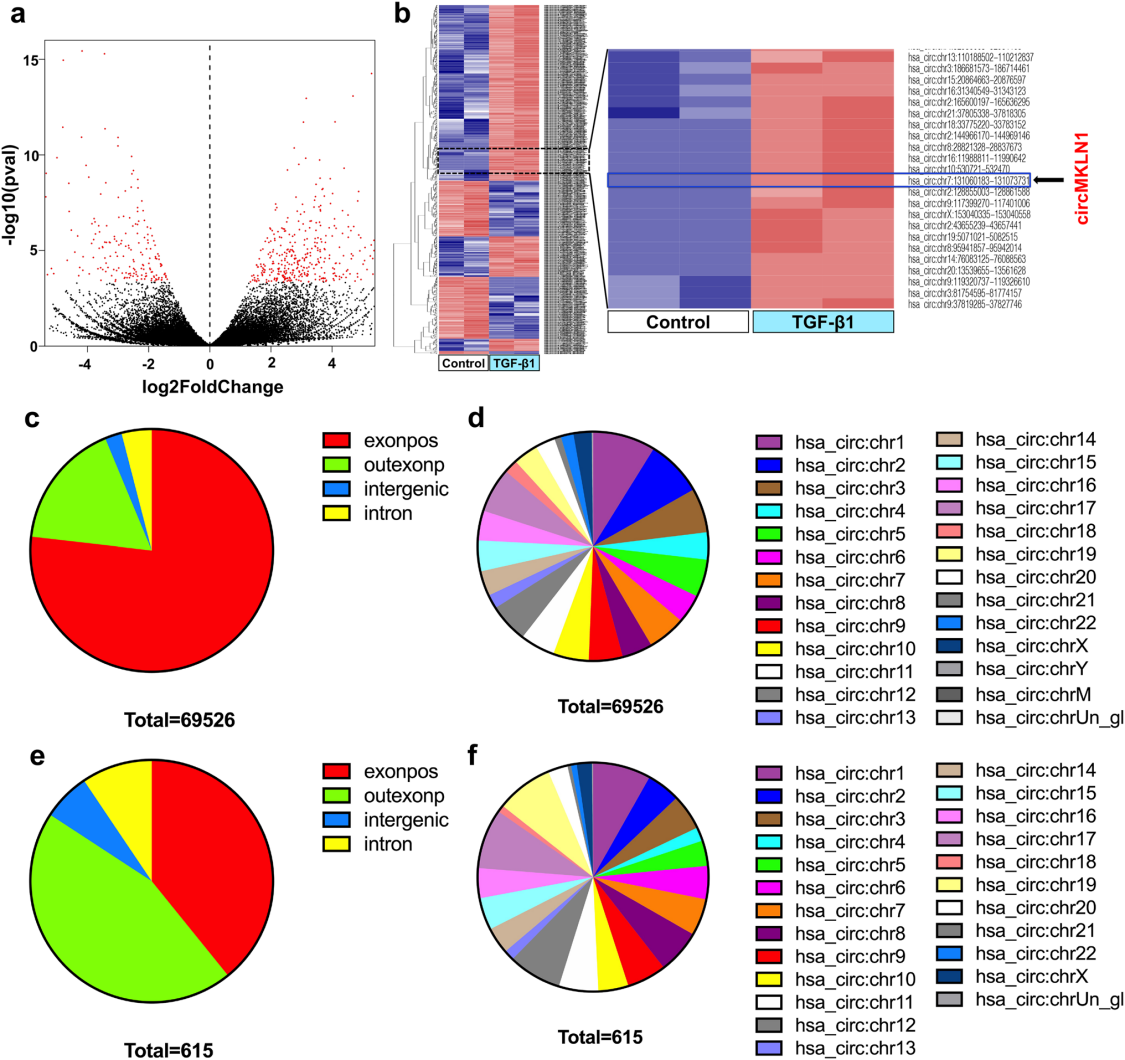
CircRNA expression profile in TGF-β1-treated AECs by high-throughput sequencing. **a** Statistical volcano plot of differentially expressed circRNAs. **b** Cluster analysis map of differentially expressed circRNAs. The red box represents circRNAs with low expression, and the blue box represents circRNAs with high expression. **c** Total circRNA composition in terms of gene distribution. **d** The distribution of total circRNAs on the chromosomes. **e** Differentially expressed circRNA composition in terms of gene distribution. **f** The distribution of differentially expressed circRNAs on the chromosomes

We successfully constructed two AEC EMT models by stimulating A549 cells with TGF-β1 and PQ (Figure S1). Then, the treated A549 cells were collected and subjected to high-throughput sequencing to detect changes in the circRNA expression profiles. We identified 69526 circRNAs in the TGF-β1 group, and 93.74% (65127/69526) of the circRNAs were exonic circRNAs (Fig. 1a–d). A total of 72152 circRNAs were identified in the PQ group. The circRNAs identified in both groups were widely distributed throughout the human chromosomes (Figure S2a–d). There were 615 differentially expressed circRNAs (403 upregulated and 212 downregulated circRNAs) in the TGF-β1 group (Fig. 1e, f) and 979 differentially expressed circRNAs (310 upregulated and 669 downregulated circRNAs) in the PQ group (Figure S2e, f) compared to the control group. Most of these differentially expressed circRNAs were also exonic circRNAs, which were widely distributed in various chromosomes.

### CircMKLN1 expression was significantly elevated in AECs and lung tissues

**Fig. 2:**
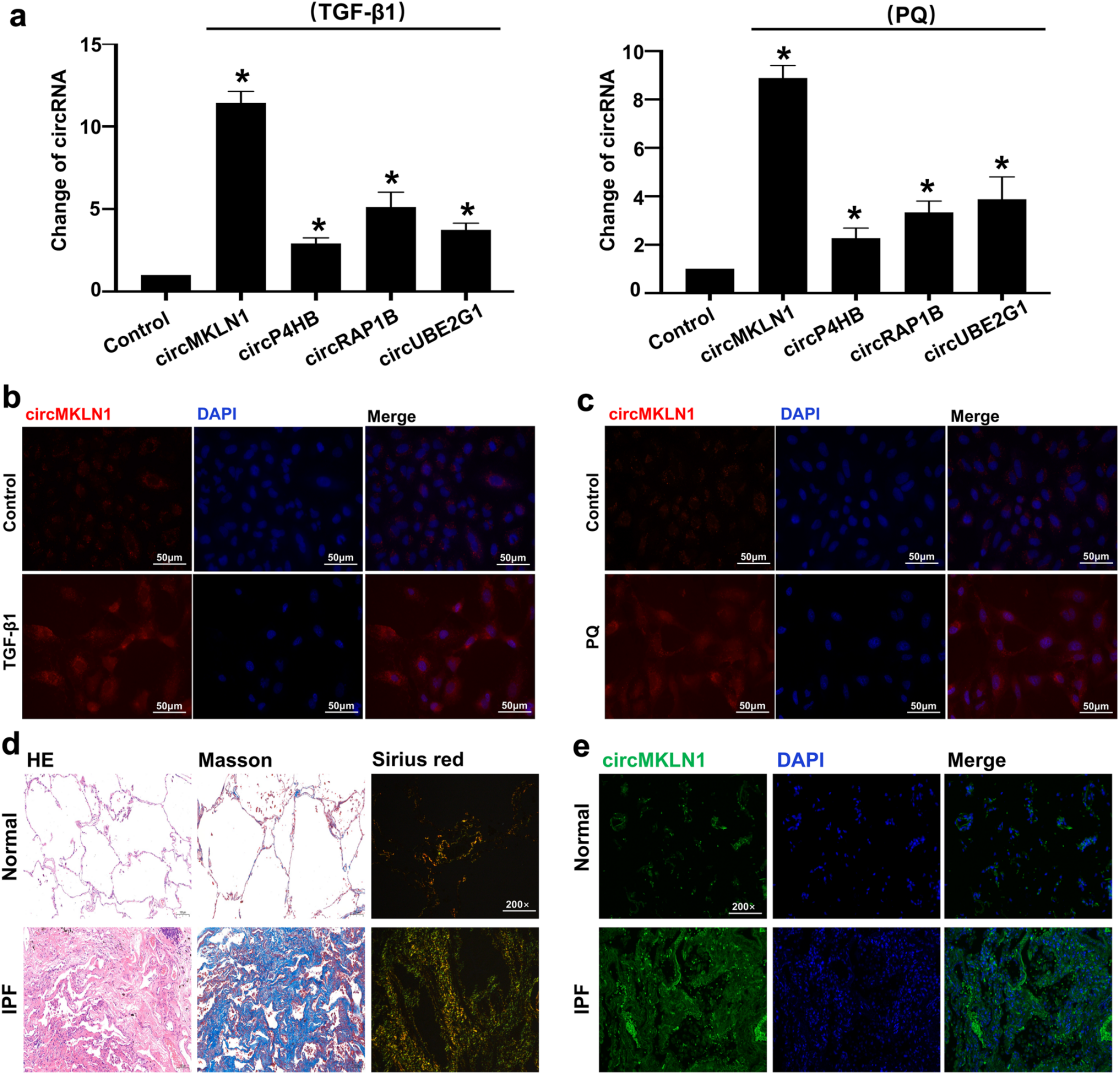
CircMKLN1 expression was significantly increased in pulmonary fibrosis. **a** The expression of circRNAs (circMKLN1, circP4HB, circRAP1B and circUBE2G1) in the TGF-β1 and PQ groups. The reference gene β-actin was used as the internal control. *p < 0.05 vs. the control group. **b** and **c** In situ hybridization assay of circMKLN1 in the TGF-β1 and PQ groups (scale bar = 50 µm). **d** HE, Masson and Sirius scarlet staining analysis of lung tissue samples from IPF patients. **e** In situ hybridization assay of circMKLN1 in lung tissue samples from IPF patients (scale bar = 200 µm)

We selected the circRNAs with a greater than fivefold elevation in both groups. Four circRNAs (circMKLN1, circP4HB, circRAP1B and circUBE2G1) were highly expressed in both groups. The expression changes of the four circRNAs were clarified by qPCR. The results showed that circMKLN1 was the most significantly elevated circRNA in both the TGF-β1 and PQ groups (Fig. 2a). Therefore, we used circMKLN1 as a target circRNA to further investigate its role and mechanism in pulmonary fibrosis. CircMKLN1 expression was significantly elevated in the TGF-β1 and PQ groups and was clustered in the cytoplasm of AECs in in situ hybridization assays (Fig. 2b, c). In lung tissue samples from IPF patients, Masson staining and Sirius scarlet staining showed a significant increase in collagen deposition. In situ hybridization experiments also showed a significant increase in circMKLN1 expression in pulmonary fibrosis patient tissues (Fig. 2d, e). The above results suggested that circMKLN1 may play an important role in pulmonary fibrosis.

### Characterization of circMKLN1

**Fig. 3:**
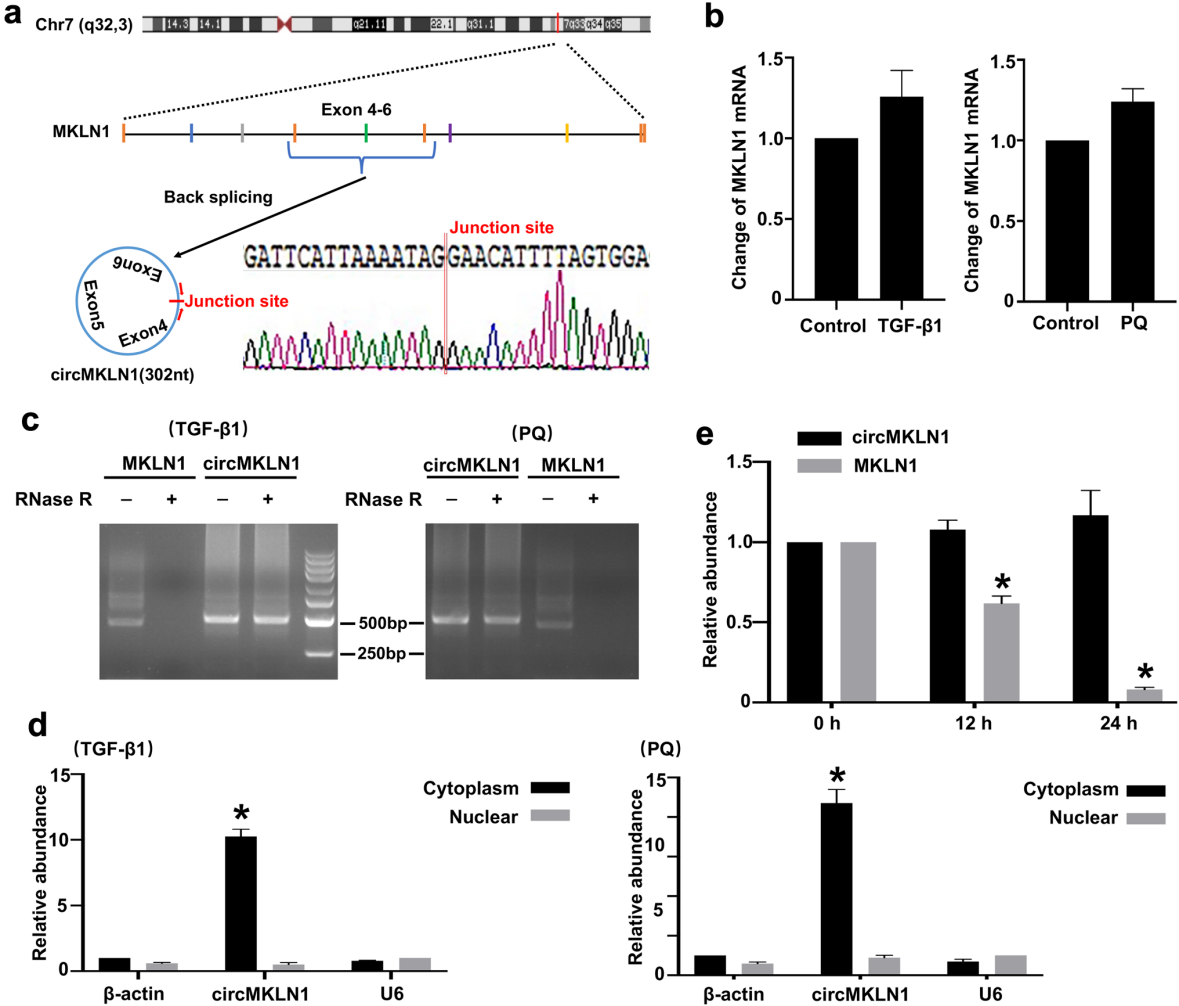
Characterization of circMKLN1. **a** Schematic diagram showing the genomic location and backsplicing pattern of circMKLN1. **b** The expression of MKLN1 mRNA in the TGF-β1 and PQ groups. The reference gene β-actin was used as the internal control. **c** Total RNA isolated from TGF-β1- or PQ-treated AECs was digested by RNase R, followed by qPCR detection of circMKLN1 and MKLN1 mRNA. **d** The expression of circMKLN1 in the cytoplasmic and nuclear fractions. **e** qPCR detection of circMKLN1 and MKLN1 mRNA expression in AECs after treatment with actinomycin D. *p < 0.05 vs. the 0 h group

CircMKLN1 is derived from chromosome 7. Sanger sequencing of the PCR products revealed that the sequencing of the circMKLN1 linkage was consistent with the high-throughput sequencing results and the sequence of the exon reverse linkage. This finding indicated that circMKLN1 was reverse sheared into a loop (Fig. 3a). There was no significant change in MKLN1 mRNA levels in either group (Fig. 3b). The stability of circMKLN1 was further examined with RNase R. A significant decrease in the expression of the linear gene MKLN1 occurred compared to that in the control group, while the expression of circMKLN1 did not change significantly after RNase R enzyme treatment. These results indicated that circMKLN1 is highly resistant to RNase R and again demonstrated that circMKLN1 is present in a circular form (Fig. 3c). The expression of circMKLN1 in the cytoplasm and nucleus was also examined. The results showed that circMKLN1 expression was significantly increased in the cytoplasm of AECs after TGF-β1 and PQ treatment, while there was no significant change in the nucleus (Fig. 3d). Addition of actinomycin D further confirmed that intracellular MKLN1 mRNA was significantly decreased, while circMKLN1 expression was not affected (Fig. 3e).

### CircMKLN1 promotes EMT progression in AECs

**Fig. 4:**
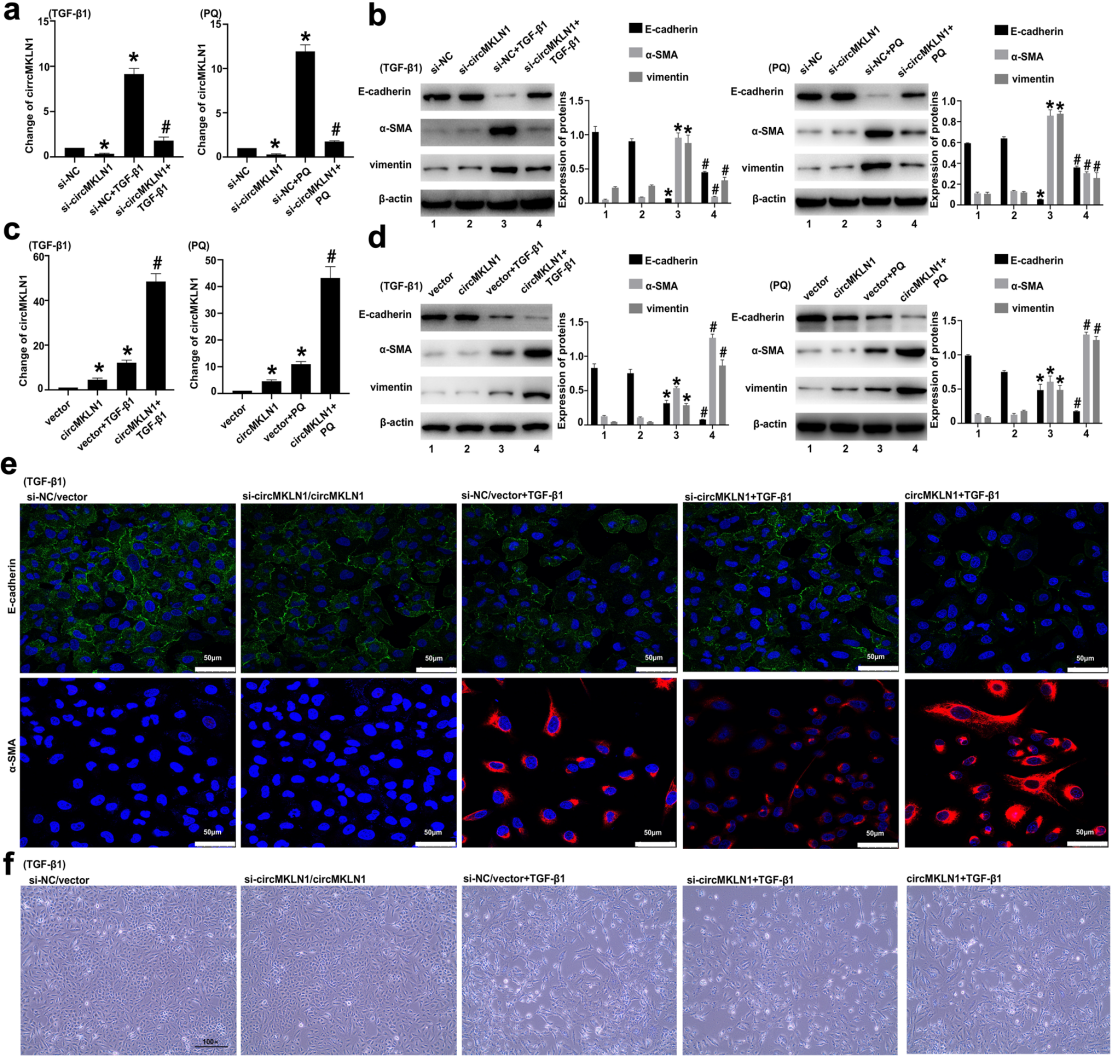
CircMKLN1 promoted EMT progression in AECs. **a** The expression of circMKLN1 in A549 cells after transfection with siRNA targeting circMKLN1. The reference gene β-actin was used as the internal control. *p < 0.05 vs. the si-NC group, #p < 0.05 vs. the si-NC + TGF-β1/PQ group. **b** The expression of EMT-related indicators (E-cadherin, α-SMA and vimentin) after inhibition of circMKLN1 in A549 cells. β-actin was used as the endogenous control. *p < 0.05 vs. the si-NC group, #p < 0.05 vs. the si-NC + TGF-β1/PQ group. **c** The expression of circMKLN1 in A549 cells after transfection with the circMKLN1 plasmid. The reference gene β-actin was used as the internal control. *p < 0.05 vs. the vector group, #p < 0.05 vs. the vector + TGF-β1/PQ group. **d** The expression of EMT-related indicators (E-cadherin, α-SMA and vimentin) after overexpression of circMKLN1 in A549 cells. β-actin was used as the endogenous control. *p < 0.05 vs. the vector group, #p < 0.05 vs. the vector + TGF-β1/PQ group. **e** Immunofluorescence analysis of E-cadherin and α-SMA in TGF-β1-treated A549 cells after inhibition or overexpression of circMKLN1 (scale bar = 50 µm). **f** Morphological changes in A549 cells treated with TGF-β1 after inhibition or overexpression of circMKLN1

We inhibited circMKLN1 expression with siRNA technology in AECs (Fig. 4a), which were then treated with TGF-β1 (for 48 h) and PQ (for 24 h). The results showed that inhibition of circMKLN1 expression induced a significant elevation of the epithelial marker E-cadherin, which was decreased by TGF-β1 and PQ, while the elevated mesenchymal markers α-SMA and vimentin were significantly inhibited (Fig. 4b). The circMKLN1 plasmid was further applied to overexpress circMKLN1 in AECs (Fig. 4c). The results showed that overexpression of circMKLN1 promoted a further decrease in the expression of E-cadherin and a further increase in the expression of α-SMA and vimentin (Fig. 4d). Immunofluorescence results also revealed that inhibition of circMKLN1 significantly attenuated the extent of TGF-β1- and PQ-induced EMT, and overexpression of circMKLN1 significantly aggravated the extent of TGF-β1- and PQ-induced EMT (Fig. 4e, Figure S3a). Morphological changes in cells were observed under an optical microscope, and the transformation of TGF-β1- and PQ-induced epithelial cells from a polygonal epithelial-like morphology to a shuttle-shaped mesenchymal morphology was positively correlated with circMKLN1 expression (Fig. 4f, Figure S3b).

### Inhibition of circMKLN1 expression in mouse lung tissue attenuates pulmonary fibrosis and EMT

**Fig. 5:**
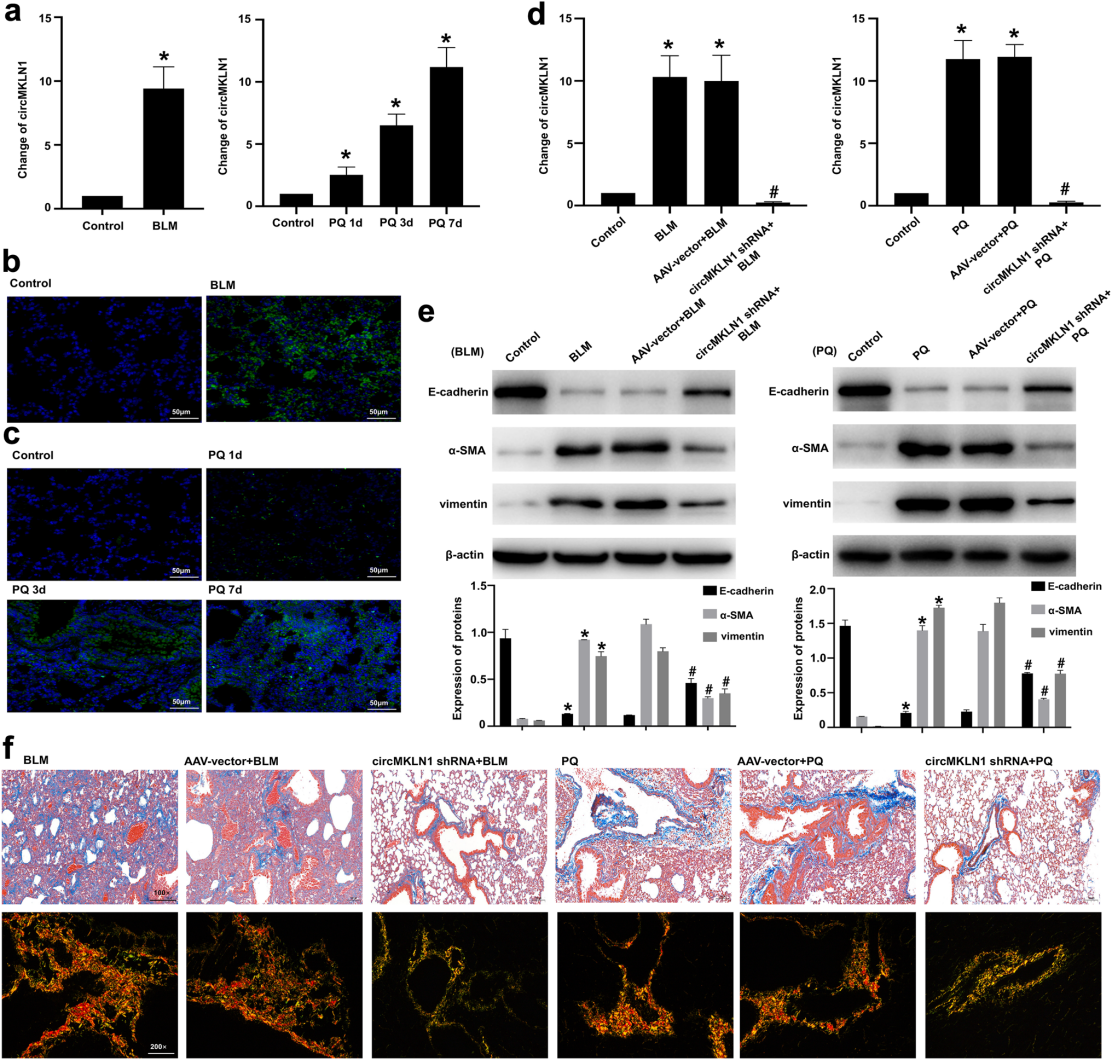
Inhibition of circMKLN1 expression in mouse lung tissue attenuated pulmonary fibrosis. **a** The expression of circMKLN1 in mouse lung tissues after treatment with BLM or PQ. The reference gene β-actin was used as the internal control. *p < 0.05 vs. the control group. **b** and **c** In situ hybridization assay of circMKLN1 in mouse lung tissues after treatment with BLM or PQ (scale bar = 50 µm). **d** Inhibition of circMKLN1 in mouse lung tissues with circMKLN1 AAV-shRNA. *p < 0.05 vs. the control group, #p < 0.05 vs. the AAV-vector + BLM/PQ group. **e** The expression of EMT-related indicators (E-cadherin, α-SMA and vimentin) after inhibition of circMKLN1 in mouse lung tissues. β-actin was used as the endogenous control. *p < 0.05 vs. the control group, #p < 0.05 vs. the AAV-vector + BLM/PQ group. **f** Masson and Sirius scarlet staining detected circMKLN1 inhibition in mouse lung tissues

We constructed two mouse lung fibrosis models with BLM and PQ to treat C57BL/6 mice. The results showed that both BLM and PQ treatment significantly increased collagen deposition in mouse lung tissues and promoted fibrotic changes in lung tissues (Figure S4a–d). Both BLM and PQ significantly decreased the expression of the epithelial marker E-cadherin and significantly increased the expression of the mesenchymal markers α-SMA and vimentin in mouse lung tissues (Figure S4e). qPCR and in situ hybridization experiments showed that the expression of circMKLN1 was significantly increased in mouse lung tissues (Fig. 5a–c). We further applied circMKLN1 adenovirus-shRNA to suppress circMKLN1 expression in mouse lung tissues (Fig. 5d). Inhibition of circMKLN1 expression in mouse lung tissues resulted in significant elevation of the epithelial marker E-cadherin, which was decreased by BLM or PQ, and significant inhibition of the mesenchymal markers α-SMA and vimentin, which were elevated (Fig. 5e). Masson staining and Sirius scarlet staining showed that inhibition of circMKLN1 significantly improved fibrotic changes in lung tissue and reduced collagen deposition in mouse lung tissue (Fig. 5f, g). We found that inhibition of circMKLN1 in mouse lung tissue also reduced lung tissue injury by HE staining (Figure S5).

### CircMKLN1 functions as a miR-26a/b sponge

**Fig. 6:**
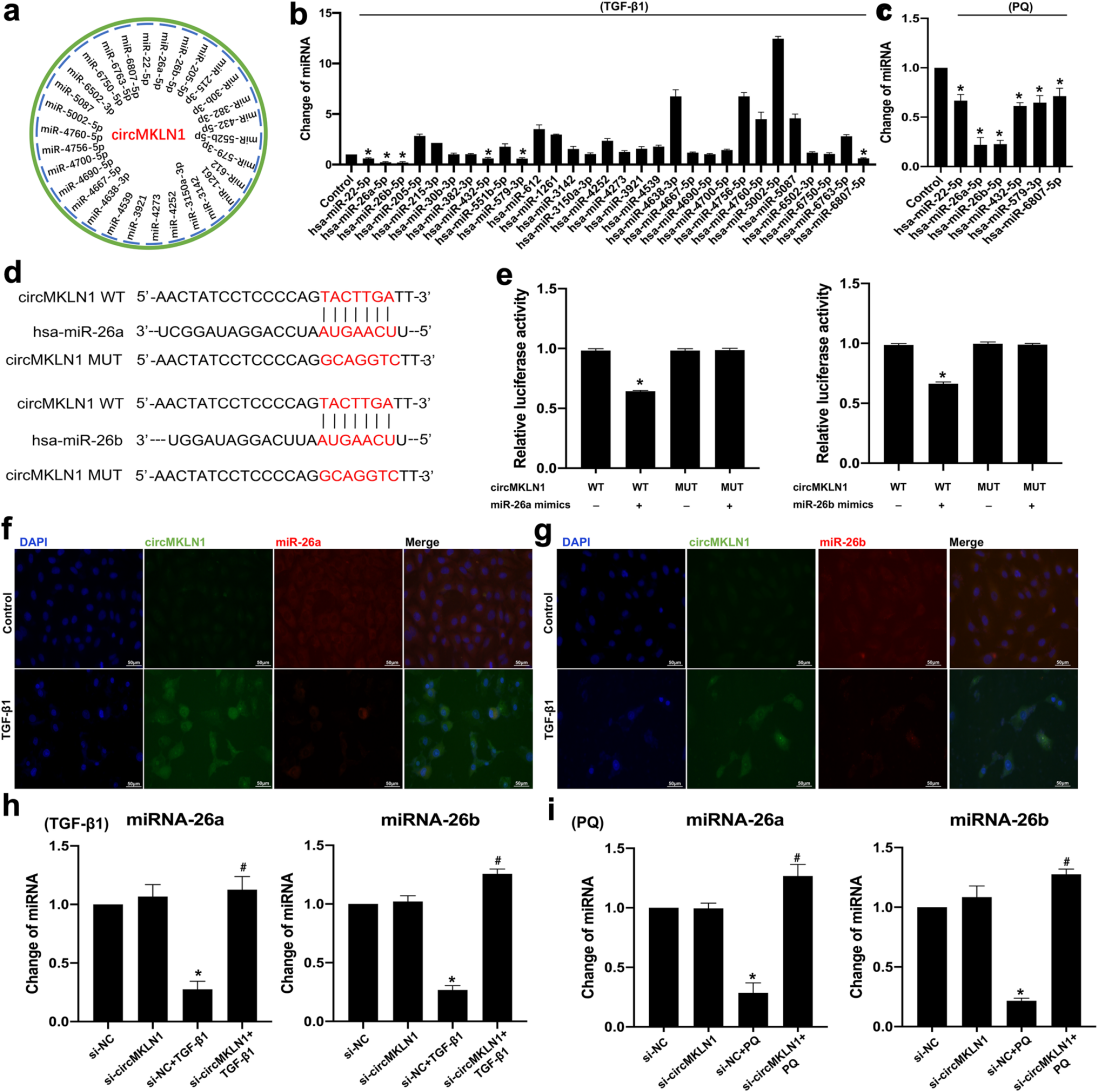
CircMKLN1 functioned as a miR-26a/b sponge. **a** Predicted miRNAs interacting with circMKLN1. **b** The expression of the predicted miRNAs combined with circMKLN1 in the TGF-β1 group. *p < 0.05 vs. the control group. **c** The expression of differentially expressed miRNAs combined with circMKLN1 in the PQ group. *p < 0.05 vs. the control group. **d** Schematic of miR-26a/b sites in circMKLN1. **e** Luciferase assays of HEK293 cells co-transfected with a scrambled control, miR-26a/b mimic, and a luciferase reporter plasmid containing wild-type circMKLN1. *p < 0.05 vs. the circMKLN1 WT group. **f** and **g** In situ hybridization assay of miR-26a/b and circMKLN1 in TGF-β1-treated A549 cells (scale bar = 50 µm). **h** and **i** The expression of miR-26a/b after inhibition of circMKLN1 in A549 cells. *p < 0.05 vs. the si-NC group, #p < 0.05 vs. the si-NC + TGF-β1/PQ group

Reports have shown that circRNAs mainly sponge miRNAs, which in turn promotes high expression of downstream target genes of miRNAs (J. Zhang et al. 2022; Zheng et al. 2016). Bioinformatics analysis showed that circMKLN1 may competitively bind approximately 30 miRNAs (Fig. 6a). qPCR results further confirmed that six miRNAs (miR-22, miR-26a, miR-26b, miR-432, miR-579, miR-6807) showed a significant decrease in TGF-β1-stimulated AECs (Fig. 6b). We further verified the changes in the above six miRNAs in PQ-stimulated AECs. qPCR results showed that miR-26a and miR-26b were the most significantly decreased (Fig. 6c). Therefore, we chose miR-26a-5p/miR-26b-5p (hereinafter miR-26a/b) as possible target miRNAs for circMKLN1 binding to further explore their mechanisms. We confirmed that miR-26a/b is a downstream target of circMKLN1 by constructing a plasmid with circMKLN1 with a miR-26 a/b binding site mutant and applying a luciferase reporter gene assay (Fig. 6d, e). The application of in situ hybridization in AECs in vitro to simultaneously detect changes in circMKLN1 and miR-26a/b expression showed cytoplasmic colocalization of intracellular circMKLN1 and miR-26a/b expression (Fig. 6f, g, Figure S6). After inhibition of circMKLN1 expression in the TGF-β1- or PQ-stimulated AEC model, miR-26a/b expression was significantly elevated (Fig. 6h, i).

### CircMKLN1 promotes EMT by upregulating the expression of the miR-26a/b target CDK8

**Fig. 7:**
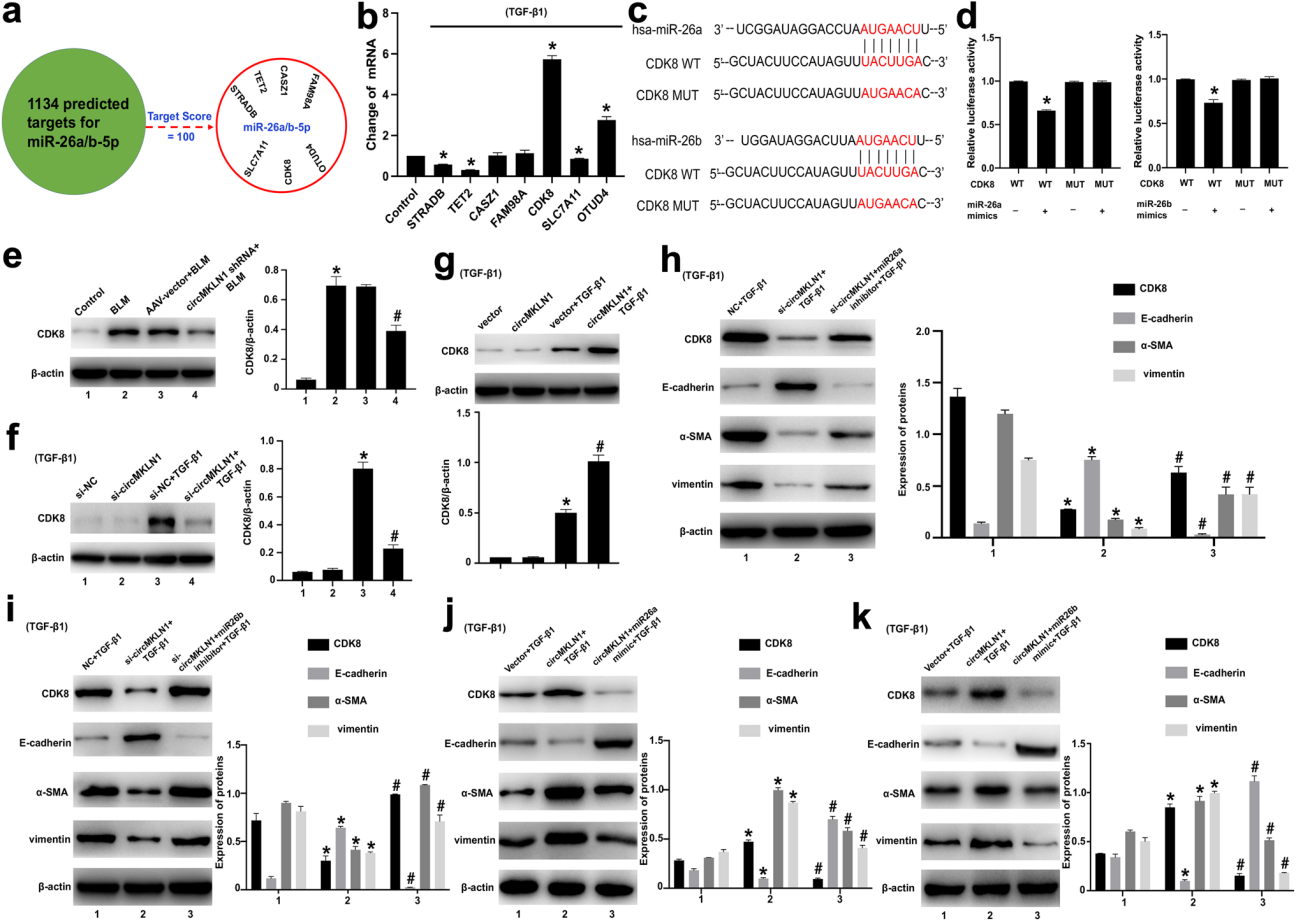
CircMKLN1 promotes EMT by upregulating the expression of the miR-26a/b target CDK8. **a** Predicted targets combined with miR-26a/b. **b** The expression of predicted targets combined with miR-26a/b in the TGF-β1 group. *p < 0.05 vs. the control group. **c** Schematic of miR-26a/b combined with CDK8. **d** Luciferase assays of HEK293 cells co-transfected with a scrambled control, miR-26a/b mimic, and a luciferase reporter plasmid containing wild-type CDK8. *p < 0.05 vs. the CDK8 WT group. **e** The level of CDK8 protein in mouse lung tissues with inhibition of circMKLN1 expression. β-actin was used as the endogenous control. *p < 0.05 vs. the control group, #p < 0.05 vs. the AAV-vector + BLM group. **f**, **g** The level of CDK8 protein in A549 cells with regulation of circMKLN1 expression. β-actin was used as the endogenous control. *p < 0.05 vs. the si-NC/vector group, #p < 0.05 vs. the si-NC/vector + TGF-β1 group. **h**, **i** The levels of CDK8 and EMT-related indicators (E-cadherin, α-SMA and vimentin) after cotransfection of circMKLN1 siRNA and miR-26a/b inhibitor in TGF-β1-treated A549 cells. β-actin was used as the endogenous control. *p < 0.05 vs. the NC + TGF-β1 group, #p < 0.05 vs. the si-circMKLN1 + TGF-β1 group. **j**, **k** The levels of CDK8 and EMT-related indicators (E-cadherin, α-SMA and vimentin) after cotransfection of the circMKLN1 plasmid and miR-26a/b mimic in TGF-β1-treated A549 cells. β-actin was used as the endogenous control. *p < 0.05 vs. the vector + TGF-β1 group, #p < 0.05 vs. the circMKLN1 + TGF-β1 group

The predicted target genes of miR-26a/b were screened separately in the miRDB database (http://mirdb.org/miRDB/), and those with the highest prediction scores (STRADB, TET2, CASZ1, FAM98A, CDK8, SLC7A11, OTUD4) were selected for further validation (Fig. 7a). CDK8 expression was the most significantly elevated in TGF-β1-stimulated AECs, and thus, CDK8 may be a miR-26a/b downstream regulatory target gene (Fig. 7b). CDK8 is a transcriptional isoform member of the cyclin-dependent kinase (CDK) family and has a more selective contribution to the regulation of gene expression levels involved in certain signaling pathways (Chou et al. 2020; Li et al. 2017). In studies of pancreatic cancer, CDK8 was found to activate the expression of factors critical for stimulating the EMT process (Snail1 and ZEB1) through the Wnt/β-catenin signaling pathway and to increase the invasion and migration of pancreatic cancer cells (Xu et al. 2015). We further applied luciferase reporter gene experiments to confirm that CDK8 is a miR-26a/b downstream target gene (Fig. 7c, d). After inhibition of circMKLN1 expression in the BLM-treated mouse lung tissue, CDK8 expression was significantly decreased (Fig. 7e). CDK8 expression was also significantly decreased after inhibition of circMKLN1 expression in AECs in vitro (Fig. 7f), while overexpression of circMKLN1 further increased CDK8 expression (Fig. 7g). We also confirmed these results in the PQ-treated mice and AECs (Figure S7a–c). Further inhibition of circMKLN1 in AECs together with inhibition of miR-26a or miR-26b partially reversed the decreased CDK8 expression and exacerbated TGF-β1- and PQ-induced EMT (Fig. 7h, i, Figure S7d). Overexpression of circMKLN1 in AECs along with miR-26a or miR-26b significantly inhibited the increased expression of CDK8 and ameliorated TGF-β1- and PQ-induced EMT (Fig. 7j, k, Figure S7e). The above results indicated that circMKLN1 increased CDK8 expression through sponge adsorption of miR-26a/b and promoted the development of EMT.

## Discussion

**Fig. 8:**
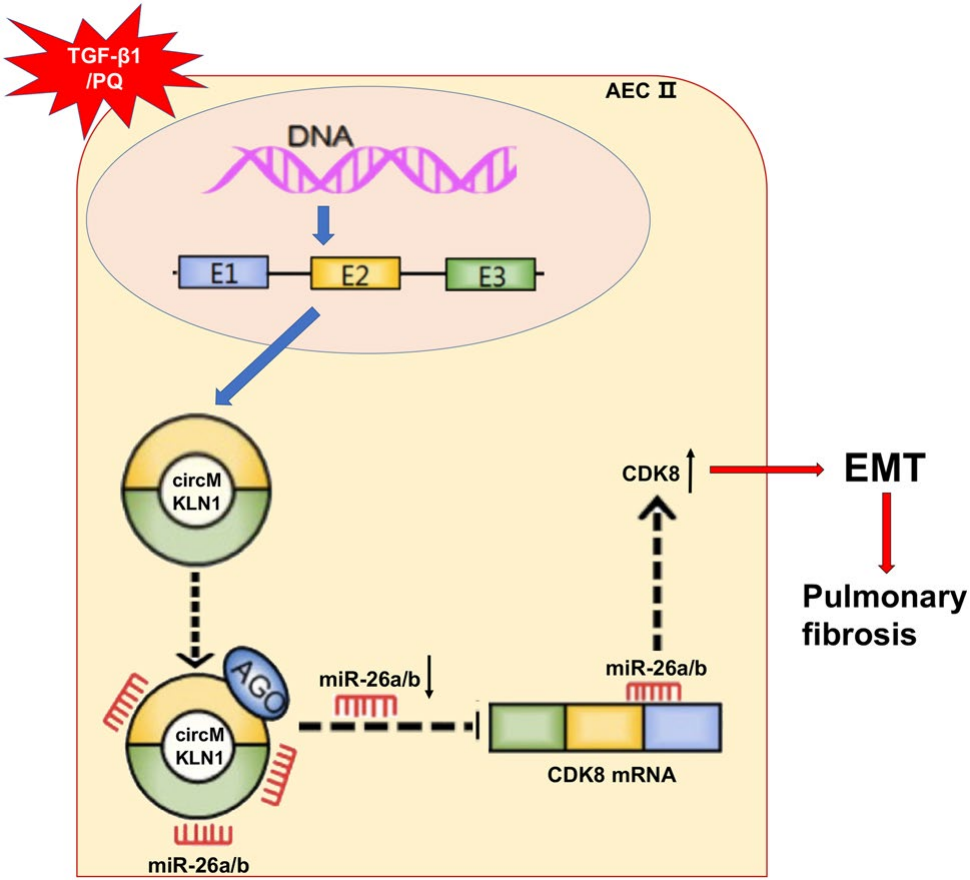
Diagram of the mechanism by which circMKLN1 promotes pulmonary fibrosis. The expression of circMKLN1 was increased in pulmonary fibrosis and promoted the EMT of AECs. The mechanism of circMKLN1 in this process might involve sponging miR-26a/b and upregulating CDK8

Pulmonary fibrosis is a progressive disease that involves initial deposition of fibrous connective tissue, which in turn affects alveolar gas exchange, leading to respiratory failure and eventually death (George et al. 2019). In vivo lineage tracing experiments have demonstrated that cells expressing mesenchymal markers have an epithelial origin in pulmonary fibrosis (Kim et al. 2006). Current studies suggest that repeated damage to type II AECs (AEC2) is an important initiating event of pulmonary fibrosis. Damaged AEC2s could trigger abnormal epithelial-fibroblast interactions through the EMT process, which induces formation of fibroblast foci and substantial extracellular matrix deposition (Katzen and Beers 2020). Thus, EMT of AEC2s is a key step in the pathogenesis of pulmonary fibrosis. In the present study, we revealed a novel mechanism by which circMKLN1 acts as a miR-26a/b sponge to promote CDK8 expression in the EMT process of pulmonary fibrosis (Fig. 8). To our knowledge, this is the first study to illustrate the expression profile of circRNAs in the AEC2 of pulmonary fibrosis. This finding may support circMKLN1 as a new intervention target for EMT in pulmonary fibrosis.

CircRNA has been a hot topic in RNA research in recent years and is a potential therapeutic target and diagnostic biomarker for certain diseases. In current studies, some circRNAs have been found to be associated with pulmonary fibrosis, but they are still in the preliminary stage of research, and their mechanisms have not been fully elucidated (Stachowiak et al. 2023; Zhou et al. 2022). In smoking-induced pulmonary fibrosis, circRNA_0026344 was shown to target exosomal miR-21 to activate SMAD7, which involves abnormal crosstalk between epithelial cells and fibroblasts (Bai et al. 2021). However, the expression profile changes and functions of circRNAs in AECs still need to be further explored. In this study, we used two models to construct an alveolar EMT model and applied high-throughput sequencing to detect changes in circRNA expression profiles. The sequencing and experimental validation results showed that circMKLN1 expression was most significantly elevated in AECs (A549 cells). In addition, we preliminarily demonstrated that circMKLN1 expression was also significantly elevated in both TGF-β1- and PQ-treated human primary type II alveolar epithelial cells, and to a similar extent as in A549 cells (Figure S8). We also confirmed the role of circMKLN1 in promoting pulmonary fibrosis and EMT.

MKLN1 is the host gene of circMKLN1. MKLN1 (Muskelin) is an intracellular kelch repeat protein consisting of discoidin, LisH, CTLH and kelch repeat structural domains. MKLN1 is involved in the regulation of cell adhesion and cytoskeletal dynamics (Adams et al. 1998; Huffman et al. 2019; Kim et al. 2014). Moreover, mutant alleles of MKLN1 may be associated with the genetic mechanism of primary chemoresistance in pediatric acute myeloid leukemia (McNeer et al. 2019). However, only a few studies have reported the role of circMKLN1 in these diseases. In prostate cancer, circMKLN1 was significantly associated with patient prognosis and has good potential for biomarker application (Hansen et al. 2022). In retinoblastoma, circMKLN1 could play a tumor suppressive role, and the mechanism may be through sponge adsorption of miR-425-5p, which in turn promotes PDCD4 and inhibits proliferation, migration and invasion of tumor cells (Li et al. 2022; Xu et al. 2021). In this study, we demonstrated that inhibition of circMKLN1 significantly reduced pulmonary fibrosis and EMT in vivo and in vitro. We revealed miRNAs (miR-26a-5p/miR-26b-5p) that share common binding sites with circMKLN1 and CDK8 in bioinformatics analyses. We confirmed circMKLN1 and miR-26a/b colocalization in the cytoplasm of AECs by FISH. A luciferase reporter assay demonstrated that miR-26a/b was a target of circMKLN1. These data suggested that circMKLN1 depended on inhibition of miR-26a/b to promote EMT.

Cyclin-dependent kinases (CDKs) are a class of serine/threonine (Ser/Thr) protein kinases that are functionally dependent on binding various cell cycle proteins (Malumbres 2014). CDKs are involved in a variety of biological processes in the body, such as metabolism, neuronal differentiation, hematopoiesis, angiogenesis, and stem cell self-renewal. Approximately 20 CDKs are known, among which CDKl-CDK6 are associated with cell cycle control; CDK7-CDK9, CDKl2 and CDKl3 are involved in transcriptional processes; and CDK8 regulates transcriptional processes by binding to mediator complexes or phosphorylating related transcription factors (Solaki and Ewald 2018). Related studies have reported that overexpressed CDK8 contributes to the development of colorectal, breast and hematologic malignancies by activating Wnt-β-catenin signaling (Witalisz-Siepracka et al. 2018; Xu et al. 2015). In the Wnt/β-catenin signaling pathway, also known as the classical Wnt signaling pathway, β-catenin enters the nucleus and binds to the TCF1/LEF1 family of transcription factors to initiate the transcription of downstream target genes (Snail1, ZEB1, etc.) and promote EMT, as shown in our study and other previous studies (Das et al. 2019; Zhu et al. 2016). Lin et al*.* found that LINC01224 adsorbed miR-193a-5p to target CDK8, promoted gastric cancer cell viability, migration and invasion, accelerated tumor formation, and promoted the expression of EMT-related proteins (Sun et al. 2021). Our luciferase reporter assay results confirmed that CDK8 is a common downstream target of miR-26a/b. Upregulated expression of circMKLN1 led to persistent activation of CDK8-induced EMT in AECs. These results suggested that circMKLN1 might promote EMT by combining with miR-26a/b and increasing the expression of CDK8.

Overall, our study found and confirmed that circMKLN1 promotes the progression of EMT in pulmonary fibrosis. We revealed a novel pathway by which circMKLN1 sponges miR-26a/b and upregulates CDK8. Our findings indicated that circMKLN1 may be a potential biomarker and therapeutic target in patients with pulmonary fibrosis. These results also provide novel insight into the development and progression of pulmonary fibrosis.

### Supplementary Information

Below is the link to the electronic supplementary material.

Supplementary file1 (DOCX 14173 KB)

## Data Availability

The data that support the findings of this study are available from the corresponding author upon request for purposes of reproducing the results or replicating the procedure.

## References

[CR1] Adams JC, Seed B, Lawler J (1998). Muskelin, a novel intracellular mediator of cell adhesive and cytoskeletal responses to thrombospondin-1. EMBO J.

[CR2] Bai J, Deng J, Han Z, Cui Y, He R, Gu Y, Zhang Q (2021). CircRNA_0026344 via exosomal miR-21 regulation of Smad7 is involved in aberrant cross-talk of epithelium-fibroblasts during cigarette smoke-induced pulmonary fibrosis. Toxicol Lett.

[CR3] Canestaro WJ, Forrester SH, Raghu G, Ho L, Devine BE (2016). Drug treatment of idiopathic pulmonary fibrosis: systematic review and network meta-analysis. Chest.

[CR4] Chanda D, Otoupalova E, Smith SR, Volckaert T, De Langhe SP, Thannickal VJ (2019). Developmental pathways in the pathogenesis of lung fibrosis. Mol Aspects Med.

[CR5] Chen T, You Y, Jiang H, Wang ZZ (2017). Epithelial-mesenchymal transition (EMT): a biological process in the development, stem cell differentiation, and tumorigenesis. J Cell Physiol.

[CR6] Chou J, Quigley DA, Robinson TM, Feng FY, Ashworth A (2020). Transcription-associated cyclin-dependent kinases as targets and biomarkers for cancer therapy. Cancer Discov.

[CR7] Das V, Bhattacharya S, Chikkaputtaiah C, Hazra S, Pal M (2019). The basics of epithelial-mesenchymal transition (EMT): a study from a structure, dynamics, and functional perspective. J Cell Physiol.

[CR8] dos Santos CC, Han B, Andrade CF, Bai X, Uhlig S, Hubmayr R, Liu M (2004). DNA microarray analysis of gene expression in alveolar epithelial cells in response to TNFalpha, LPS, and cyclic stretch. Physiol Genomics.

[CR9] Foster KA, Oster CG, Mayer MM, Avery ML, Audus KL (1998). Characterization of the A549 cell line as a type II pulmonary epithelial cell model for drug metabolism. Exp Cell Res.

[CR10] George PM, Patterson CM, Reed AK, Thillai M (2019). Lung transplantation for idiopathic pulmonary fibrosis. Lancet Respir Med.

[CR11] Hansen EB, Fredsoe J, Okholm TL, Ulhoi BP, Klingenberg S, Jensen JB, Sorensen KD (2022). The transcriptional landscape and biomarker potential of circular RNAs in prostate cancer. Genome Med.

[CR12] Huffman N, Palmieri D, Coppola V (2019). The CTLH complex in cancer cell plasticity. J Oncol.

[CR13] Katzen J, Beers MF (2020). Contributions of alveolar epithelial cell quality control to pulmonary fibrosis. J Clin Invest.

[CR14] Kim KK, Kugler MC, Wolters PJ, Robillard L, Galvez MG, Brumwell AN, Chapman HA (2006). Alveolar epithelial cell mesenchymal transition develops in vivo during pulmonary fibrosis and is regulated by the extracellular matrix. Proc Natl Acad Sci U S A.

[CR15] Kim KH, Hong SK, Hwang KY, Kim EE (2014). Structure of mouse muskelin discoidin domain and biochemical characterization of its self-association. Acta Crystallogr D Biol Crystallogr.

[CR16] Kristensen LS, Hansen TB, Veno MT, Kjems J (2018). Circular RNAs in cancer: opportunities and challenges in the field. Oncogene.

[CR17] Li M, Zhao X, Liu Y, An J, Xiao H, Wang C (2017). Aberrant expression of CDK8 regulates the malignant phenotype and associated with poor prognosis in human laryngeal squamous cell carcinoma. Eur Arch Otorhinolaryngol.

[CR18] Li R, Wang Y, Song X, Sun W, Zhang J, Liu Y, Lv C (2018). Potential regulatory role of circular RNA in idiopathic pulmonary fibrosis. Int J Mol Med.

[CR19] Li X, Yang L, Chen LL (2018). The biogenesis, functions, and challenges of circular RNAs. Mol Cell.

[CR20] Li F, Yin YK, Zhang JT, Gong HP, Hao XD (2022). Role of circular RNAs in retinoblastoma. Funct Integr Genomics.

[CR21] Malumbres M (2014). Cyclin-dependent kinases. Genome Biol.

[CR22] McNeer NA, Philip J, Geiger H, Ries RE, Lavallee VP, Walsh M, Kentsis A (2019). Genetic mechanisms of primary chemotherapy resistance in pediatric acute myeloid leukemia. Leukemia.

[CR23] Nieto MA, Huang RY, Jackson RA, Thiery JP (2016). Emt: 2016. Cell.

[CR24] Parimon T, Yao C, Stripp BR, Noble PW, Chen P (2020). Alveolar epithelial type II cells as drivers of lung fibrosis in idiopathic pulmonary fibrosis. Int J Mol Sci.

[CR25] Qi F, Li Y, Yang X, Wu Y, Lin L, Liu X (2020). Hsa_circ_0044226 knockdown attenuates progression of pulmonary fibrosis by inhibiting CDC27. Aging.

[CR26] Qu S, Yang X, Li X, Wang J, Gao Y, Shang R, Li H (2015). Circular RNA: a new star of noncoding RNAs. Cancer Lett.

[CR27] Raghu G, Remy-Jardin M, Richeldi L, Thomson CC, Inoue Y, Johkoh T, Wilson KC (2022). Idiopathic pulmonary fibrosis (an update) and progressive pulmonary fibrosis in adults: an official ATS/ERS/JRS/ALAT clinical practice guideline. Am J Respir Crit Care Med.

[CR28] Solaki M, Ewald JC (2018). Fueling the cycle: CDKs in carbon and energy metabolism. Front Cell Dev Biol.

[CR29] Stachowiak Z, Narozna B, Szczepankiewicz A (2023). Non-coding RNAs in pulmonary diseases: comparison of different airway-derived biosamples. Int J Mol Sci.

[CR30] Sun H, Yan J, Tian G, Chen X, Song W (2021). LINC01224 accelerates malignant transformation via MiR-193a-5p/CDK8 axis in gastric cancer. Cancer Med.

[CR31] Tan S, Gou Q, Pu W, Guo C, Yang Y, Wu K, Peng Y (2018). Circular RNA F-circEA produced from EML4-ALK fusion gene as a novel liquid biopsy biomarker for non-small cell lung cancer. Cell Res.

[CR32] Thannickal VJ, Toews GB, White ES, Lynch JP, Martinez FJ (2004). Mechanisms of pulmonary fibrosis. Ann Rev Med.

[CR33] Wang J, Zhu M, Pan J, Chen C, Xia S, Song Y (2019). Circular RNAs: a rising star in respiratory diseases. Respir Res.

[CR34] Witalisz-Siepracka A, Gotthardt D, Prchal-Murphy M, Didara Z, Menzl I, Prinz D, Sexl V (2018). NK cell-specific CDK8 deletion enhances antitumor responses. Cancer Immunol Res.

[CR35] Xu W, Wang Z, Zhang W, Qian K, Li H, Kong D, Tang Y (2015). Mutated K-ras activates CDK8 to stimulate the epithelial-to-mesenchymal transition in pancreatic cancer in part via the Wnt/beta-catenin signaling pathway. Cancer Lett.

[CR36] Xu L, Long H, Zhou B, Jiang H, Cai M (2021). CircMKLN1 suppresses the progression of human retinoblastoma by modulation of miR-425-5p/PDCD4 axis. Curr Eye Res.

[CR37] Yao W, Li Y, Han L, Ji X, Pan H, Liu Y, Ni C (2018). The CDR1as/miR-7/TGFBR2 axis modulates EMT in silica-induced pulmonary fibrosis. Toxicol Sci.

[CR38] Zhang XO, Wang HB, Zhang Y, Lu X, Chen LL, Yang L (2014). Complementary sequence-mediated exon circularization. Cell.

[CR39] Zhang JX, Lu J, Xie H, Wang DP, Ni HE, Zhu Y, Wang RL (2019). circHIPK3 regulates lung fibroblast-to-myofibroblast transition by functioning as a competing endogenous RNA. Cell Death Dis.

[CR40] Zhang J, Wang C, Jia C, Zhang Y, Qing X, Zhang Y, Pan Z (2022). The role of circular RNAs in the physiology and pathology of the mammalian ovary. Int J Mol Sci.

[CR41] Zheng Q, Bao C, Guo W, Li S, Chen J, Chen B, Huang S (2016). Circular RNA profiling reveals an abundant circHIPK3 that regulates cell growth by sponging multiple miRNAs. Nat Commun.

[CR42] Zhou J, Chen Y, He M, Li X, Wang R (2022). Role of circular RNAs in pulmonary fibrosis. Int J Mol Sci.

[CR43] Zhu Y, Tan J, Xie H, Wang J, Meng X, Wang R (2016). HIF-1alpha regulates EMT via the Snail and beta-catenin pathways in paraquat poisoning-induced early pulmonary fibrosis. J Cell Mol Med.

