# Supplementary material for: Circular RNA MKLN1 promotes epithelial-mesenchymal transition in pulmonary fibrosis by regulating the miR-26a/b-5p/CDK8 axis in human alveolar epithelial cells and mice models
Source: Arch Toxicol. 2024 Mar 9;98(5):1399–413. doi: 10.1007/s00204-024-03700-x (PMC10965569; doi:10.1007/s00204-024-03700-x)
Supplement: Supplementary file 1 — Supplementary file1 (DOCX 14173 KB) [file 204_2024_3700_MOESM1_ESM.docx]

**Figure S1**


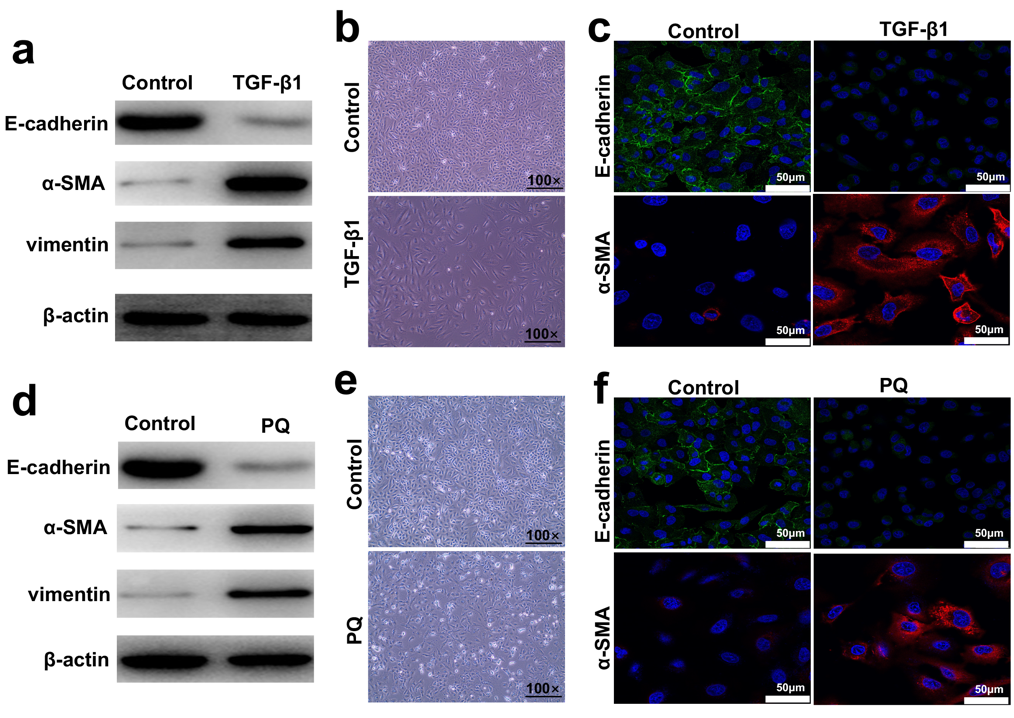


**Figure S1 Alveolar epithelial cell EMT models by stimulated with TGF-β1 and PQ to A549 cells.** **(a)** The expression of EMT related indicators (E-cadherin, α-SMA and vimentin) after TGF-β1 treated A549 cells. β-actin served as the loading control. **(b)** The morphological changes of A549 cells after TGF-β1 treatment. **(c)** Immunofluorescence analysis of E-cadherin and α-SMA in TGF-β1 treated A549 cells. (scale bar = 50 µm). **(d)** The expression of EMT related indicators (E-cadherin, α-SMA and vimentin) after PQ treated A549 cells. β-actin served as the loading control. **(e)** The morphological changes of A549 cells after PQ treatment. **(f)** Immunofluorescence analysis of E-cadherin and α-SMA in PQ treated A549 cells. (scale bar = 50 µm).

**Figure S2**


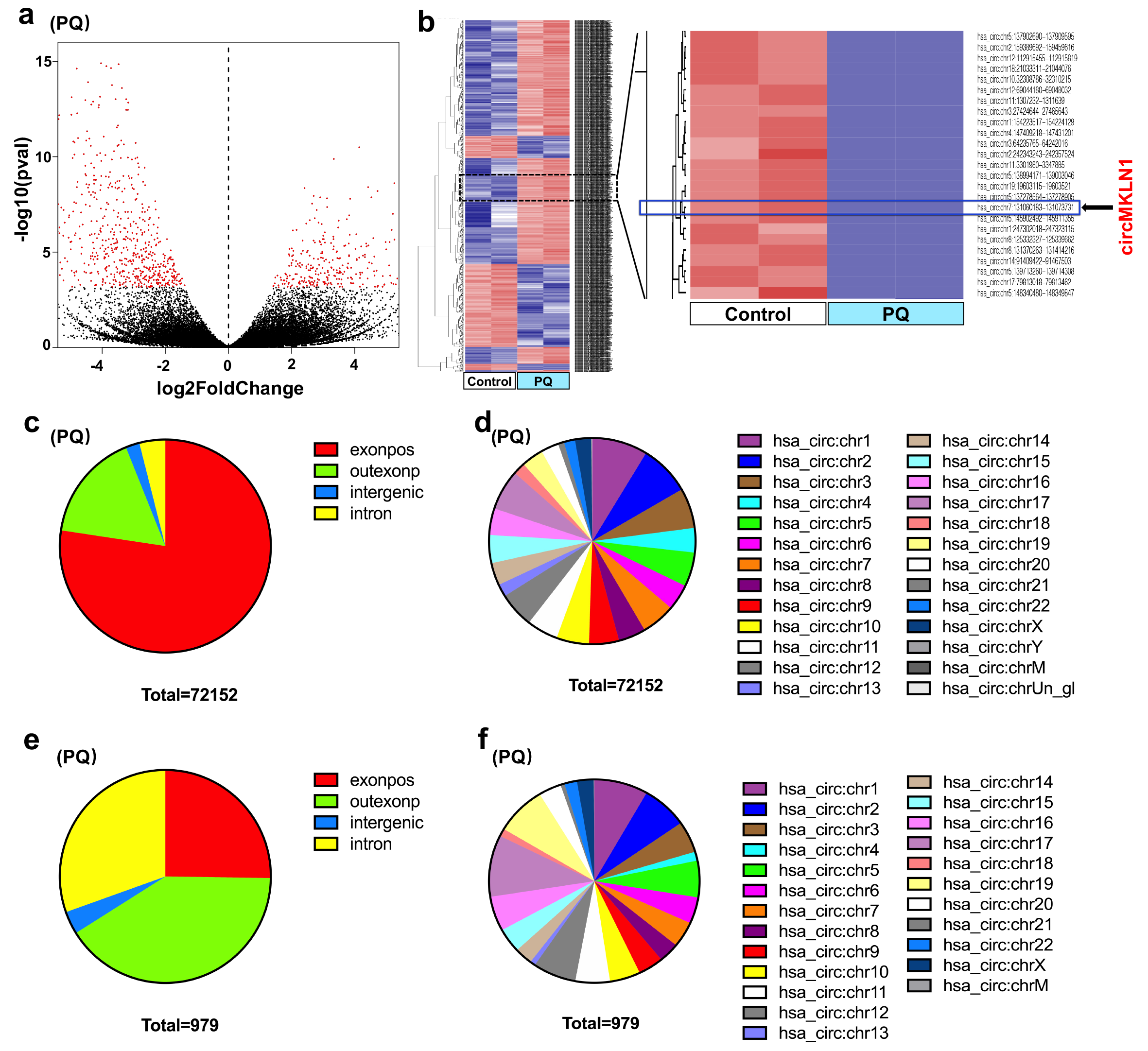


**Figure S2 CircRNAs expression profile in PQ treated alveolar epithelial cells by high-throughput sequencing.** **(a)** Statistical volcano plot of differentially expressed circRNAs. **(b)** Cluster analysis map of differentially expressed circRNAs. Red box represents low expression circRNAs and blue box represents high expression circRNAs. **(c)** Total ﻿circRNAs composition in terms of genes distribution. **(d)** ﻿The distribution of total circRNAs on the chromosomes. **(e)** Differentially expressed ﻿circRNAs composition in terms of genes distribution. **(f)** ﻿The distribution of differentially expressed circRNAs on the chromosomes.

**Figure S3**


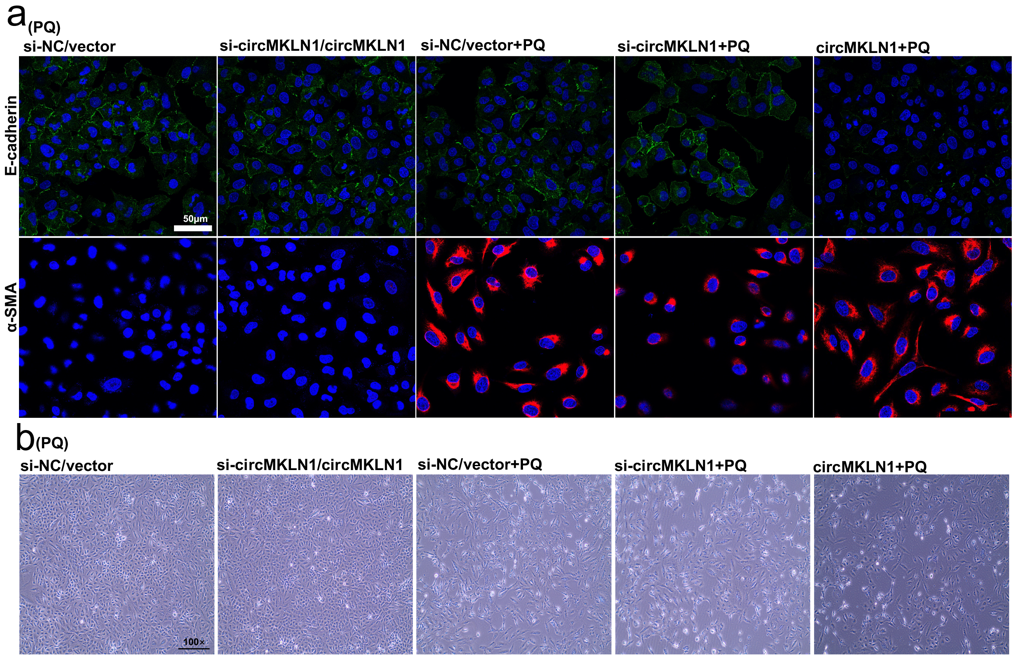


**Figure S3 CircMKLN1 regulated EMT in PQ treated alveolar epithelial cells.** **(a)** Immunofluorescence analysis of E-cadherin and α-SMA in PQ treated A549 cells after inhibited or overexpressed circMKLN1. (scale bar = 50 µm). **(b)** The morphological changes of A549 cells with PQ treatment after inhibited or overexpressed circMKLN1.

**Figure S4**


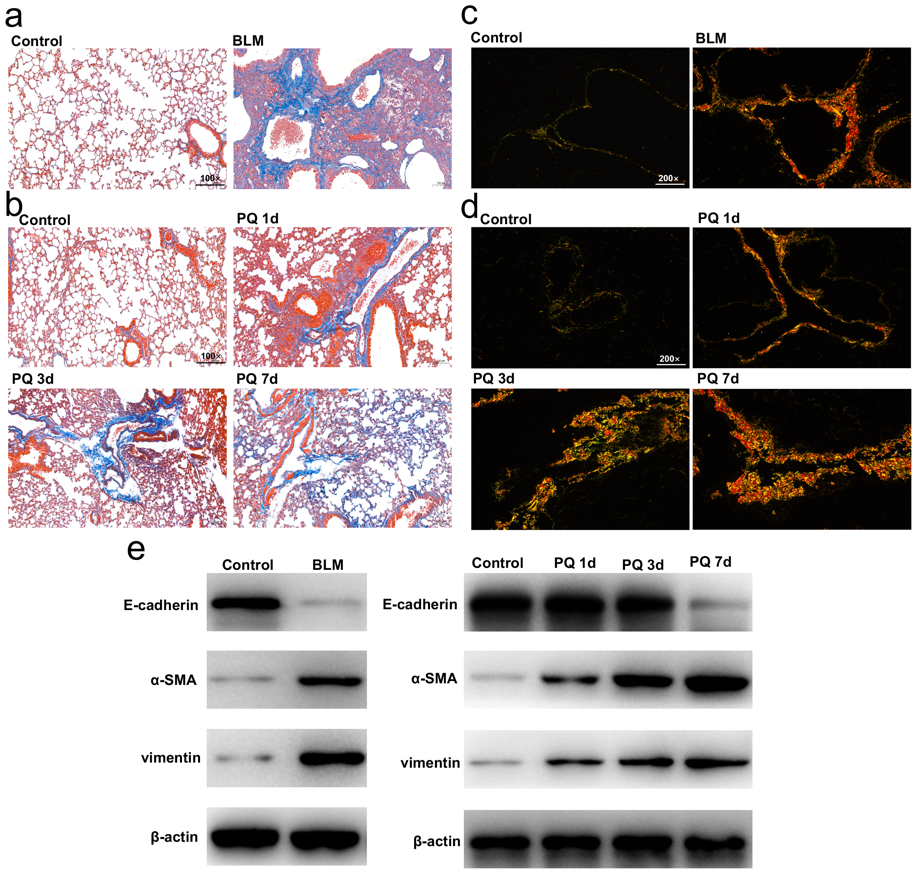


**Figure S4 BLM and PQ treatment significantly promoted fibrotic and EMT changes in mouse lung tissues**. **(a and b)** Masson staining detected the mouse lung tissues after treated with BLM and PQ. **(c and d)** Sirius scarlet staining detected the mouse lung tissues after treated with BLM and PQ. **(e)** The expression of EMT related indicators (E-cadherin, α-SMA and vimentin) in the mouse lung tissues after treated with BLM and PQ. β-actin served as the loading control.

**Figure S5**


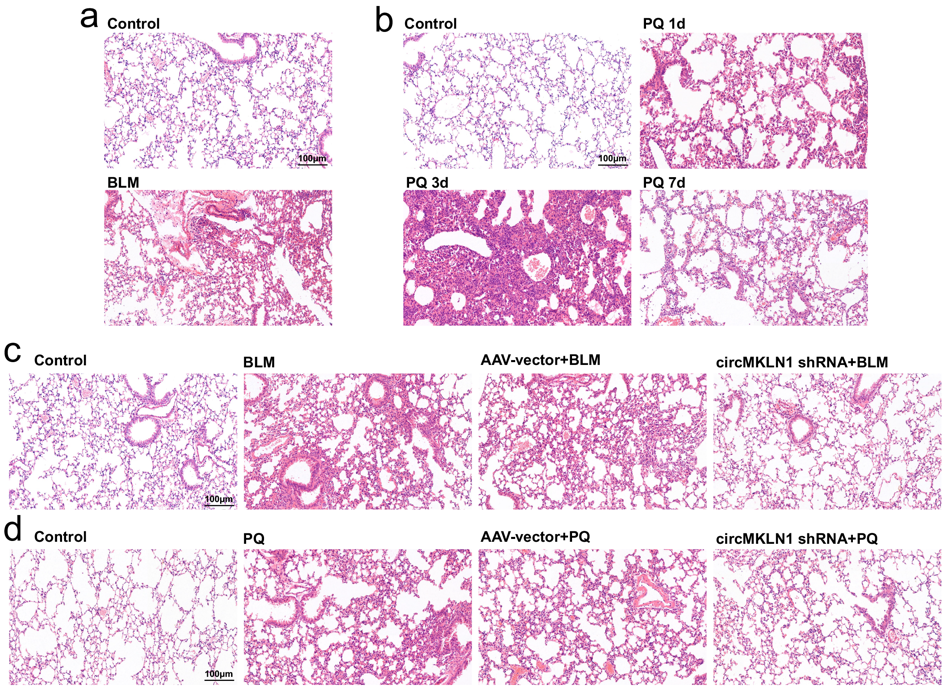


**Figure S5 HE staining the mouse lung tissues after inhibited circMKLN1 with BLM and PQ treatment.** **(a and b)** HE staining detected the mouse lung tissues after treated with BLM and PQ. **(c and d)** HE staining detected the mouse lung tissues with inhibited circMKLN1.

**Figure S6**


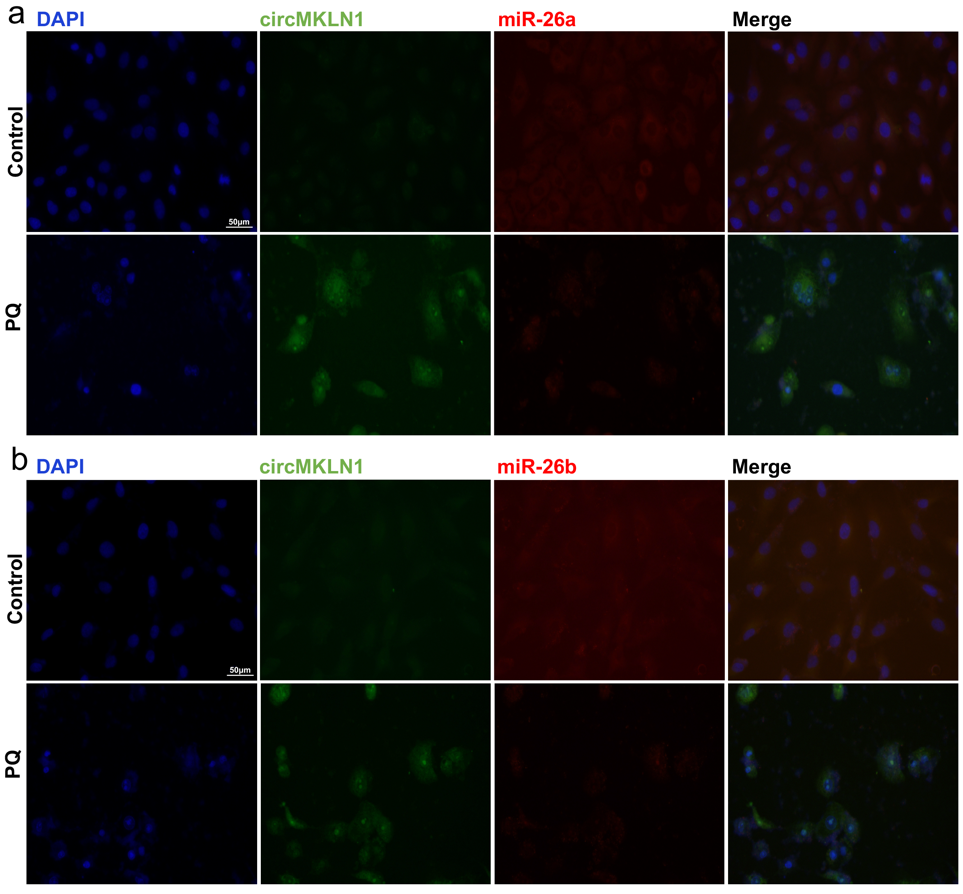


**Figure S6 CircMKLN1 and miR-26a/b expression in PQ stimulated alveolar epithelial cells**. **(a and b)** Situ hybridization assay analysis of miR-26a/b and circMKLN1 in PQ treated A549 cells. (scale bar = 50 µm).

**Figure S7**


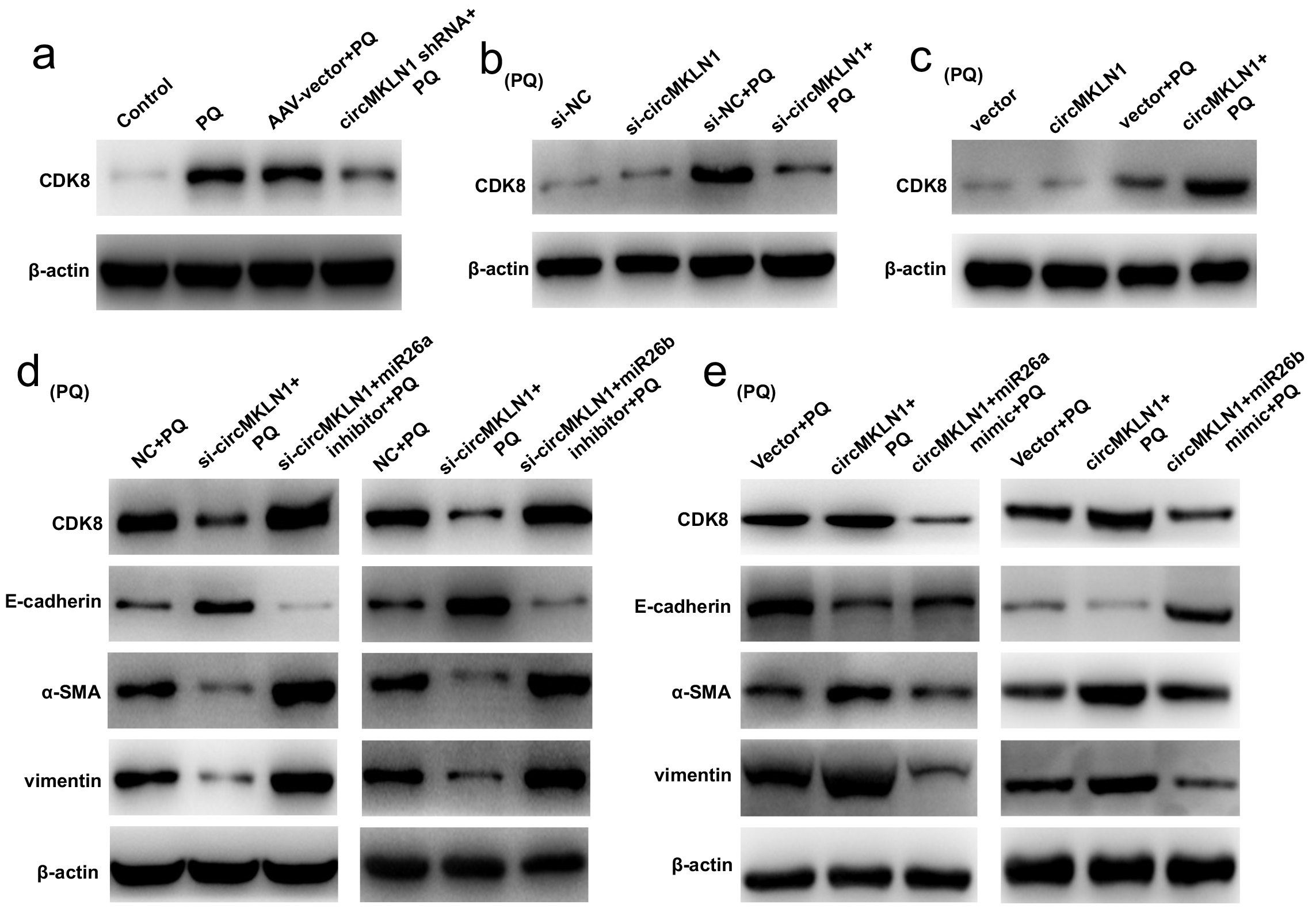


**Figure S7 CircMKLN1 promotes EMT through upregulating the expression of the miR-26a/b target CDK8 in PQ stimulated alveolar epithelial cells.** **(a-c)** The level of CDK8 proteins in mouse lung tissues and A549 cells with regulating the expression of circMKLN1 and treated with PQ. β-actin served as the loading control. **(d)** The levels of CDK8 and EMT related indicators (E-cadherin, α-SMA and vimentin) after co-transfected circMKLN1 siRNA and miR-26a/b inhibitor in PQ treated A549 cells. β-actin served as the loading control. **(e)** The levels of CDK8 and EMT related indicators (E-cadherin, α-SMA and vimentin) after co-transfected circMKLN1 plasmid and miR-26a/b mimic in PQ treated A549 cells. β-actin served as the loading control.

**Figure S8**


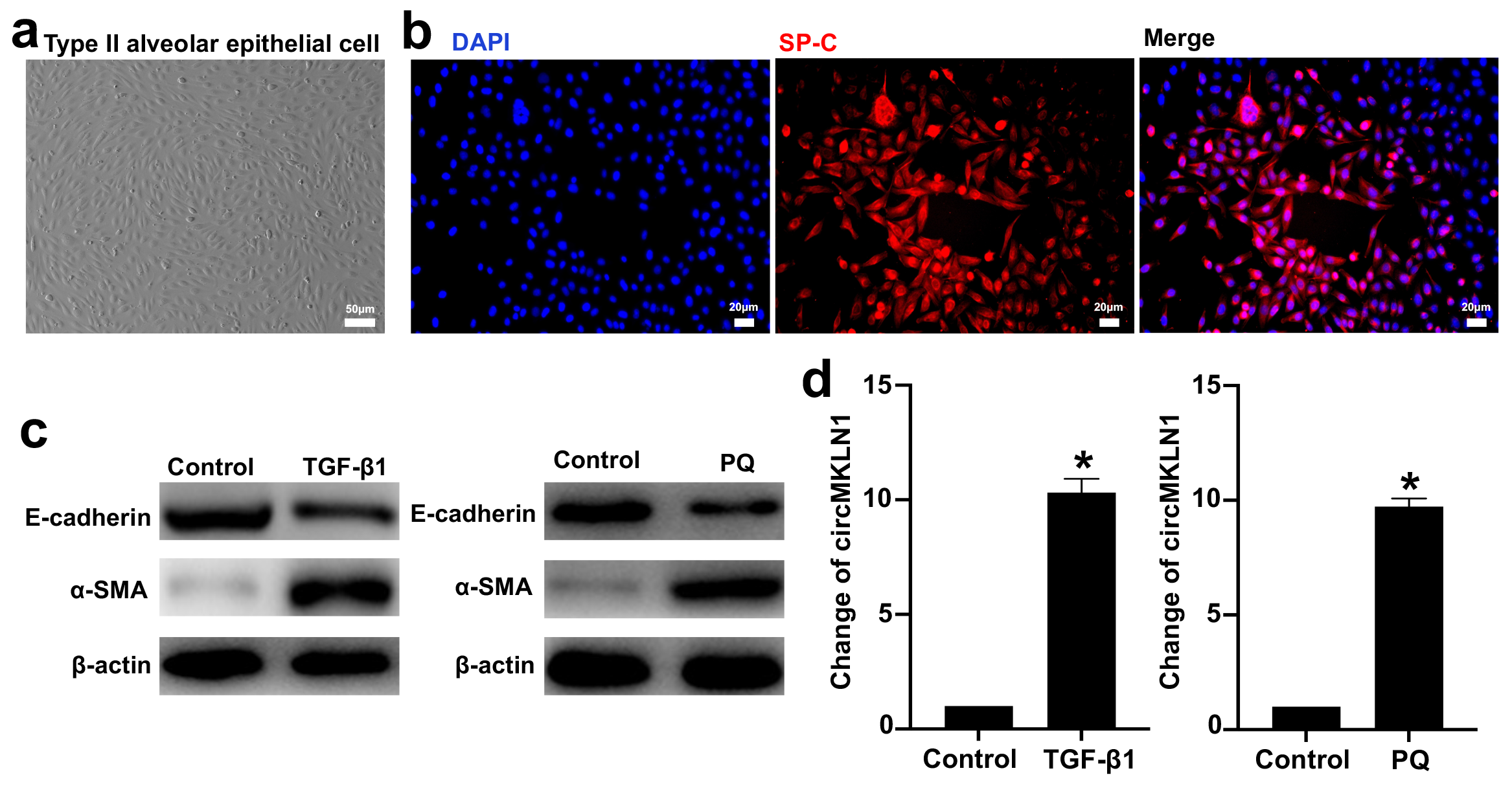


**Figure S8** The expression of circMKLN1 in human primary type II alveolar epithelial cells. The human primary type II alveolar epithelial cells were given by Shanghai Fuheng Biology. **(a)** The morphological of human primary type II alveolar epithelial cells under the phase contrast microscope (scale bar = 50 µm). **(b)** Immunofluorescence analysis of SP-C, which was the marker of type II alveolar epithelial cells (scale bar = 20 µm). **(c)** The expression of EMT-related indicators (E-cadherin and α-SMA) in human primary type II alveolar epithelial cells after treated with TGF-β1 or PQ. β-actin was used as the endogenous control. **(d)** The expression of circMKLN1 in human primary type II alveolar epithelial cells after treated with TGF-β1 or PQ. The reference gene β-actin was used as the internal control. * p < 0.05 vs. the control group.

**Table S1 The sequences of siRNA, shRNA, mimics and inhibitors**

| Species | ﻿Gene | ﻿Sequence (﻿5’-3’; ﻿3’- 5’) |
| --- | --- | --- |
| ﻿Homo | ﻿circMKLN1 | AAAUAGGAACAUUUUAGUGTT; CACUAAAAUGUUCCUAUUUTT |
| sapiens |  |  |
| siRNA | NC | UUCUCCGAACGUGUCACGUTT; ACGUGACACGUUCGGAGAATT |
| Mouse | ﻿circMKLN1 | ﻿CACTAAAATGTTCCAATTT |
| shRNA | NC | TTCTCCGAACGTGTCACGT |
| mimics | ﻿miR-26a | ﻿UUCAAGUAAUCCAGGAUAGGCU; CCUAUCCUGGAUUACUUGAAUU |
|  | miR-26b | UUCAAGUAAUUCAGGAUAGGU; CUAUCCUGAAUUACUUGAAUU |
|  | NC | UUCUCCGAACGUGUCACGUTT; ACGUGACACGUUCGGAGAATT |
| inhibitors | ﻿miR-26a | ﻿AGCCUAUCCUGGAUUACUUGAA |
|  | miR-26b | ACCUAUCCUGAAUUACUUGAA |
|  | NC | CAGUACUUUUGUGUAGUACAA |

**Table S2 The sequences of primers used in qPCR for each target genes**

|  | ﻿Gene | ﻿Primer sequence (﻿forward, 5’-3’; ﻿reverse, 3’- 5’) |
| --- | --- | --- |
| ﻿Homo | ﻿circMKLN1 | AGAGCTGTTGTCCAGTGGCT; ACTGGGGAGGATAGTTGCTCTC |
| sapiens | circP4HB | ﻿GCCATCGATGACATACCATTTG; ﻿CAGCCTCTCTGCCAGCTTCTT |
|  | circRAP1B | ﻿CGAGTTAAAGACACTGATGATACTG; ﻿GTTGTGCATCTACTTCAACTTGCTT |
|  | circUBE2G1 | ﻿AAAAGTTGCCCGCTGTGTAAGA; ﻿CCTTAAAAACACCACCTTCACCT |
|  | MKLN1 | GAGGACATCCAGTCTCGAATAG; CAAATAGGTCTTTGATCGCTGG |
|  | CDK8 | GGGATCTCTATGTCGGCATGT; CACACCTTCCTATCAGCATGAG |
|  | STRADB | GGTCACGTCCATCCACTAGAG; ACCAGTGTTCCTGTGGGAGTA |
|  | TET2 | GATAGAACCAACCATGTTGAGGG; TGGAGCTTTGTAGCCAGAGGT |
|  | CASZ1 | CCGAGGGTGTCTACATGGTG; CCCGTCCGAATCCTTCTCC |
|  | FAM98A | CTTGAGGTGAGTGGGCTACTA; TGAGGTATGTGAGCAAGAGGAG |
|  | SLC7A11 | TCTCCAAAGGAGGTTACCTGC; AGACTCCCCTCAGTAAAGTGAC |
|  | OTUD4 | TTCTGATGTGGATTACAGAGGGC; ACGCATGTTGTCTTACTCCTGA |
|  | β-actin | CTGGAACGGTGAAGGTGACA; AAGGGACTTCCTGTAACAATGCA |
| Mouse | ﻿circMKLN1 | ﻿ TGAACAGATGTTCCCTTGTCG; ﻿CGATAGCAGGCCTTTCAAGC |
|  | β-actin | TGGGAATGGGTCAGAAGGA; ATTGAGAAAGGGCGTGGC |

**Table S3 The probe sequences used in FISH**

|  | ﻿Gene | ﻿Sequence (﻿5’-3’) |
| --- | --- | --- |
| ﻿Homo | ﻿circMKLN1 | GGTT+TGTCCACTAA+AATGTTCCT+AT |
| sapiens | miR-26a-5p | ﻿AGCCT+ATCCTGGATTACT+TGAA |
|  | miR-26b-5p | ﻿ACCT+ATCCTGAATTACT+TGAA |
| Mouse | ﻿circMKLN1 | ﻿A+CTAAAATGTTC+CAATTTTAATGAA+TCGAC |
